# Supplementary figures and images for: Delivery of ceramide phosphoethanolamine lipids to the cleavage furrow through the endocytic pathway is essential for male meiotic cytokinesis
Source: PLoS Biol. 2022 Sep 28;20(9):e3001599. doi: 10.1371/journal.pbio.3001599 (PMC9550178; doi:10.1371/journal.pbio.3001599)

**Figure S1**  
**Kunduri et al., 2022**

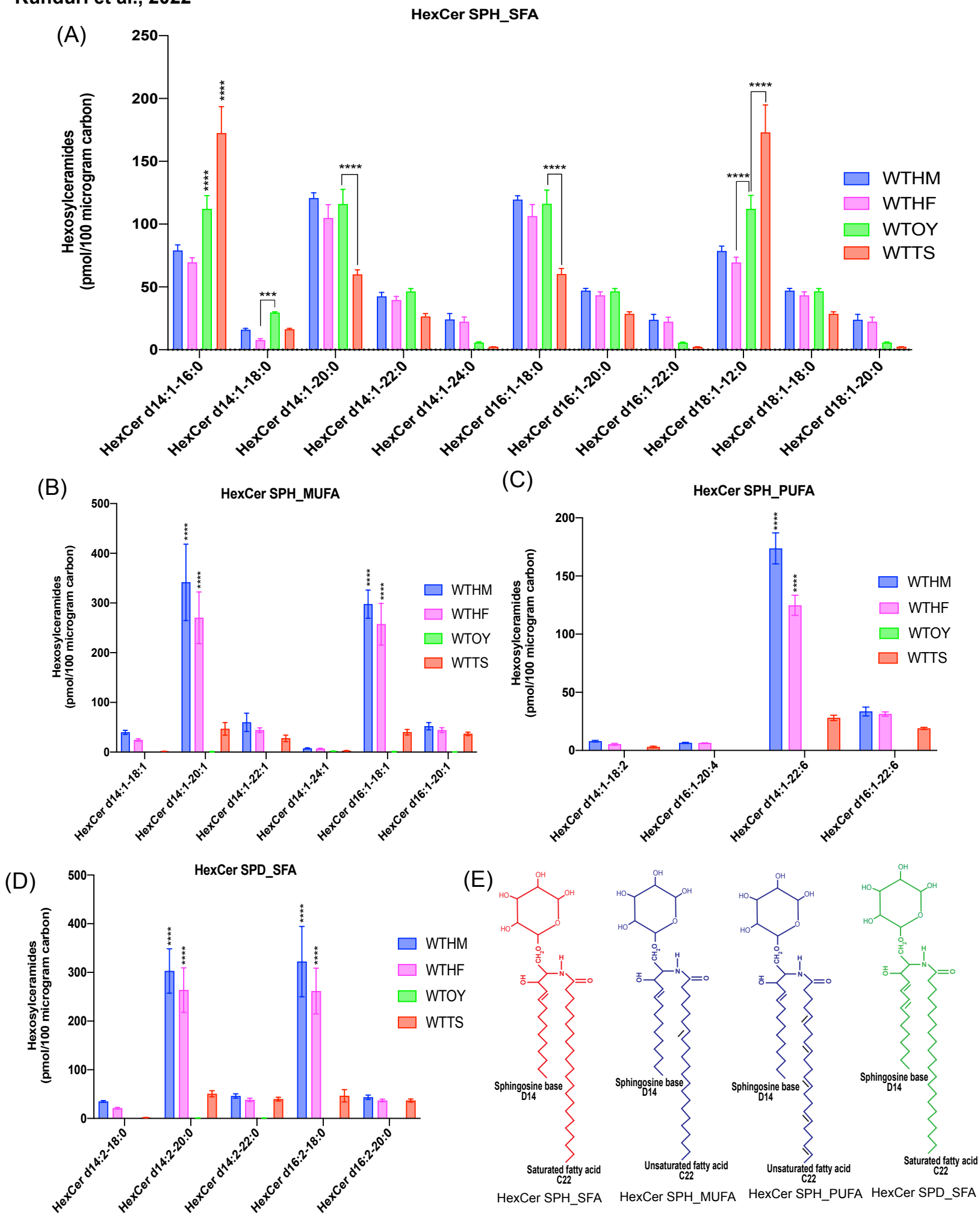

Supplement: S1 Fig — (A) Hexosylceramides composed of sphingosine base linked to saturated fatty acid (HexCer_SPH-SFA) and its subspecies are shown as picomols (pmol) in 100 micrograms of carbon. WTHM, WTHF, WTOY, and WTTS. (B) Hexosylceramide subspecies composed of sphingosine base linked to monounsaturated fatty acid (HexCer_SPH_MUFA). (C) Hexosylceramide subspecies composed of sphingosine base linked to polyunsaturated fatty acid (HexCer_SPH_PUFA). (D) Hexosylceramide subspecies composed of sphingadiene base linked to saturated fatty acid (HexCer_SPD_SFA). (E) Cartoon showing the representative chemical structure of 3 major and 1 minor HexCer subspecies including HexCer_SPH_SFA, HexCer_SPH_MUFA, HexCer_SPD_SFA, and HexCer_SPH_PUFA, respectively. Statistical significance was calculated using mean, SD, and N in Prism 8. The 2-way ANOVA multiple comparison was used to calculate p-values where **** p ≤ 0.0001, *** p ≤ 0.001, ** p ≤ 0.01, * p ≤ 0.05, and ns p >0.05. Three independent biological replicates were taken for each sample and lipids were extracted from 400 heads or 250 pairs of ovary or testis for each biological replicate. The data underlying the graphs shown in this figure can be found in S1 Data. SD, standard deviation; WTHF, wild-type heads from female; WTHM, wild-type heads from male; WTOY, wild-type ovary; WTTS, wild-type testis. (PDF) [file pbio.3001599.s001.pdf]

Figure S2  
Kunduri et al., 2022

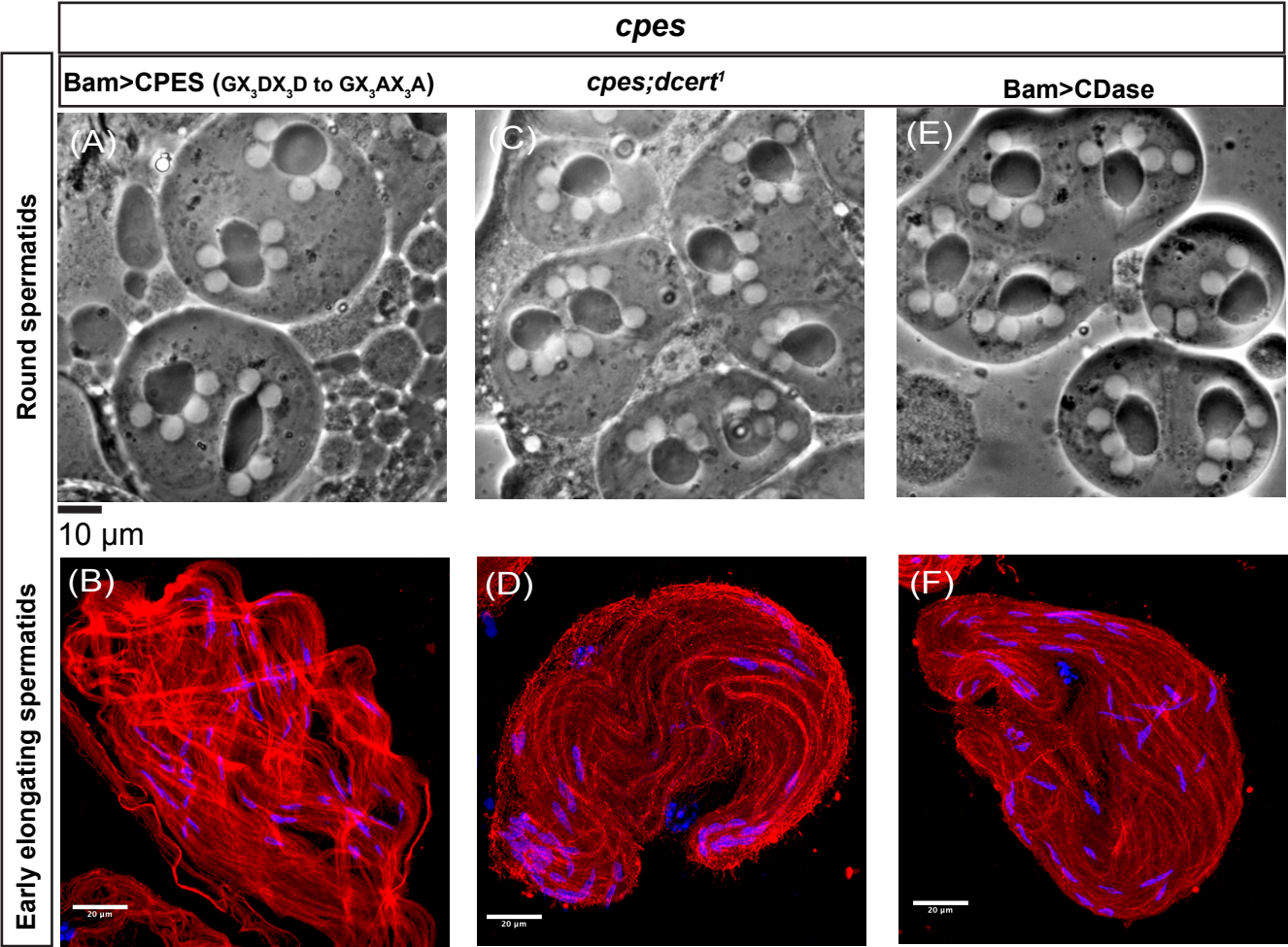

Supplement: S2 Fig — (A, C, and E) Live testis squash preparation followed by phase contrast microscopy of round spermatids; (B, D, and F) are immunofluorescence images of early elongating spermatids from samples corresponding to A, C, and E, respectively. Immunostaining was performed with beta tubulin primary antibody and Alexa Fluor 568 conjugated secondary antibody (red). Nuclei are stained with DAPI (blue) (A and B), the UAS-CPES active site mutant (GX3DX3D to GX3AX3A) was expressed using bam-Gal4 in cpes mutant background. (C and D), cpes; dcert1 double mutants. (E and F) The UAS CDase (neutral ceramidase) was expressed using bam-Gal4 driver in cpes mutant background. (PDF) [file pbio.3001599.s002.pdf]

Figure S3  
Kunduri et al., 2022

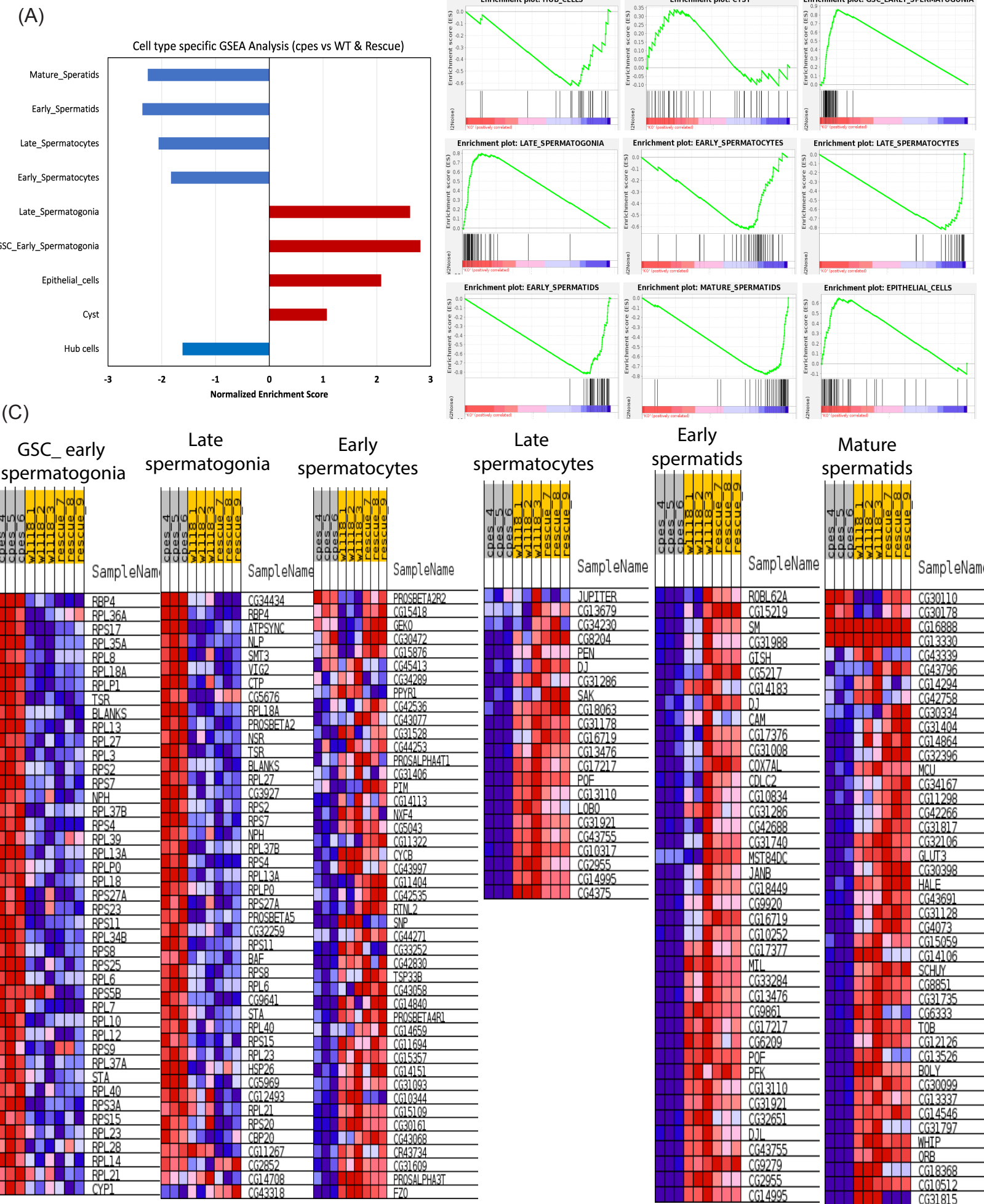

Supplement: S3 Fig — (A) GSEA using top 50 cell type-specific expressed gene sets and compared between cpes versus WT and bam-Gal4>UAS CPES rescue (Rescue). Red bars indicate gene sets enriched in cpes mutants and blue bars indicate gene sets enriched in WT and Rescue. (B) Enrichment plots for each of the cell type show the distribution of individual genes in each set (vertical lines on red to blue parallel bar). Genes with positive ES indicate that they are enriched in cpes mutants whereas negative ES indicate that they are enriched in WT and Rescue. (C) Heatmap comparison of cell type-specific enriched gene sets in WT (w1118), cpes, and Rescue (bam-Gal4>UAS CPES rescue). Red indicates gene sets enriched in cpes mutants and blue indicate gene sets enriched in WT and Rescue. The data underlying the graphs shown in this figure can be found in S2 Data. ES, enrichment score; GSEA, Gene Set Enrichment Analysis; WT, wild type. (PDF) [file pbio.3001599.s003.pdf]

Figure S4  
Kunduri et. al., 2022

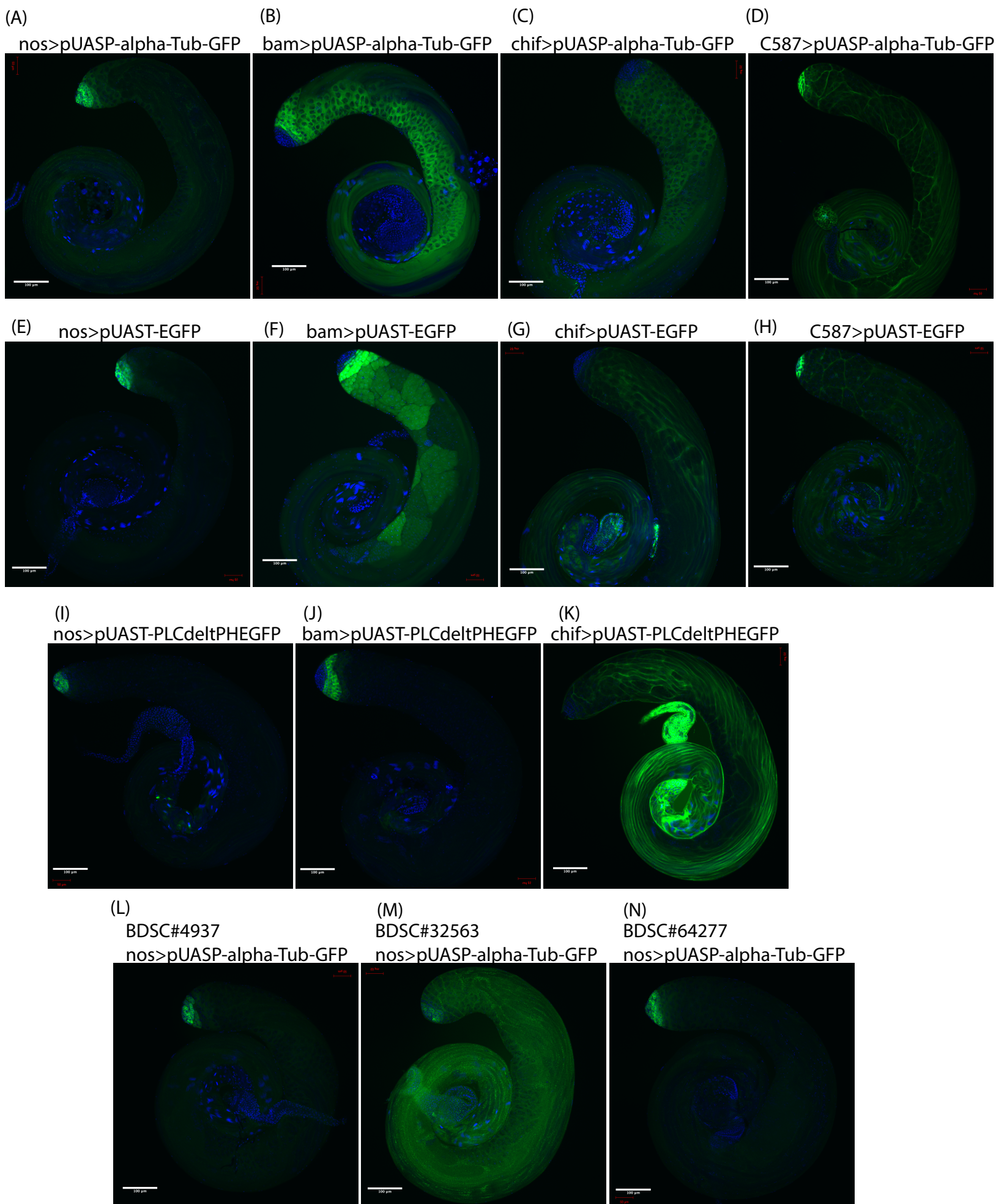

Supplement: S4 Fig — (A-) Whole mount Drosophila testis immunostained with anti-GFP antibody (green), DNA stained with DAPI (blue). (A) nanos-Gal4 (nos) drives the expression of pUASP-alpha-Tubulin-GFP, (B) bam-Gal4 drives the expression of pUASP-alpha-Tubulin-GFP, (C) chiffon-Gal4 drives the expression of alpha-Tubulin-GFP, (D) C587-Gal4 drives the expression of alpha-Tubulin-GFP, (E) nanos-Gal4 (nos) drives the expression of pUAST-EGFP, (F) bam-Gal4 drives the expression of pUAST-EGFP, (G) chiffon-Gal4 drives the expression of pUAST-EGFP, (H) C587-Gal4 drives the expression of alpha-Tubulin-GFP, (I) nanos-Gal4 (nos) drives the expression of pUAST-PLCδ-PH-EGFP, (J) bam-Gal4 drives the expression of pUAST-PLCδ-PH-EGFP, (K) chiffon-Gal4 drives the expression of pUAST-PLCδ-PH-EGFP, (L–N) verification of various nanos-Gal4 drivers and their expression pattern. BDSC#4937 (used in this study) and BDSC#64227 specifically express transgene in GSC and early spermatogonia compared to BDSC#32563 that expresses more broadly. (PDF) [file pbio.3001599.s004.pdf]

**Figure S5**  
**Kunduri et al., 2022**

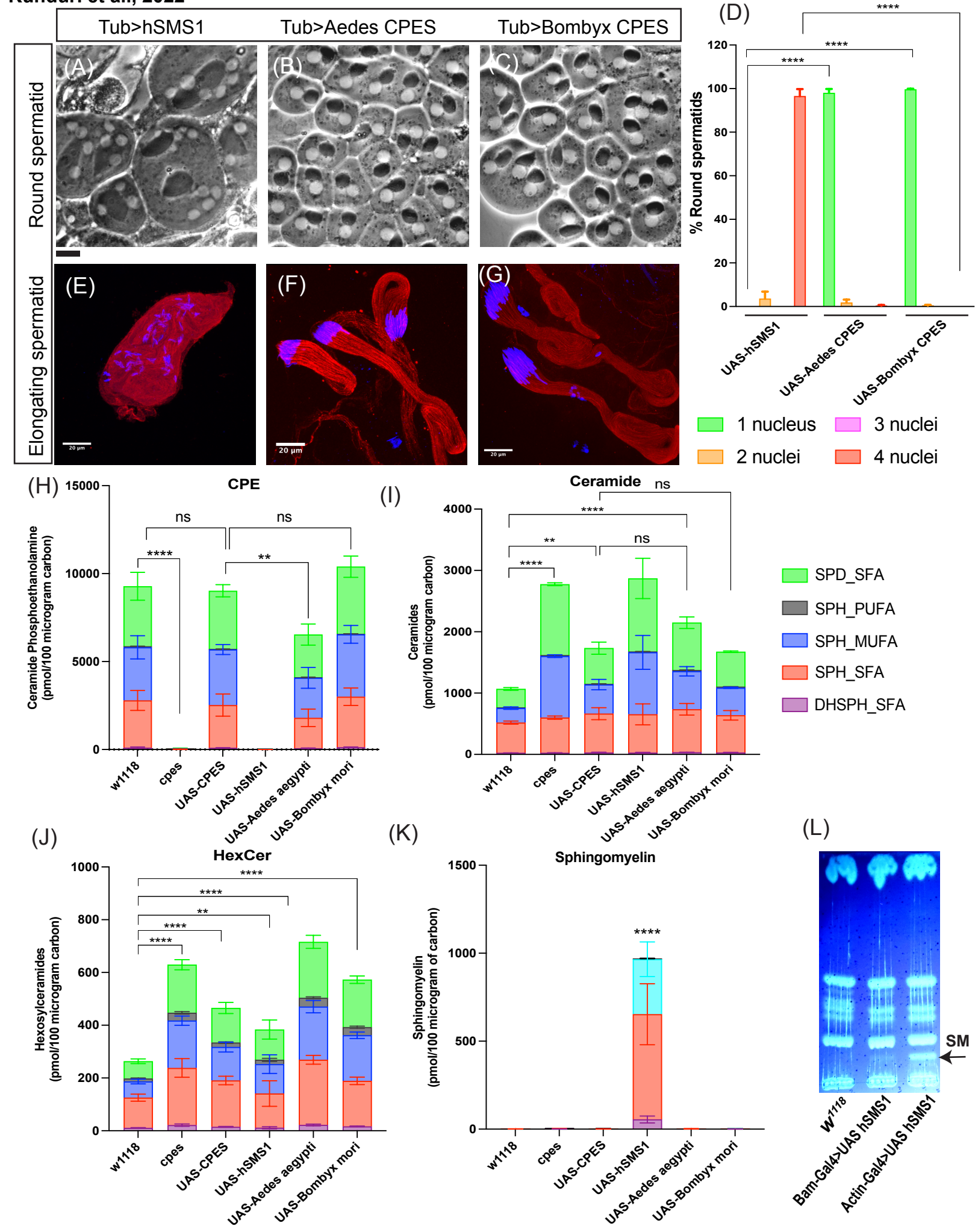

Supplement: S5 Fig — (A–C) Phase contrast microscopy of round spermatids where UAS hSMS1 (A), UAS Aedes CPES (B), and UAS Bombyx CPES (C) were ubiquitously expressed in cpes mutant background using tubulin Gal4. The scale bar is equivalent to 10 μm. (D) Measurement of cytokinetic defects in UAS-hSMS1, UAS-Aedes aegypti, and UAS-Bombyx mori CPES rescue testes; 4 independent experiments for UAS-hSMS1 (round spermatid count, n = 500), 2 independent experiments for UAS-Aedes aegypti (round spermatid count, n = 1,100), and 4 independent experiments for UAS-Bombyx CPES (round spermatid count, n = 1,900). (E–G) Confocal images of elongating spermatids immunostained with beta tubulin primary antibody and Alexa Fluor 568 secondary antibody (red) and DAPI for DNA (blue). UAS hSMS1 (E), UAS Aedes CPES (F), and UAS Bombyx CPES (G). (H–K) Sphingolipid analysis of lipids extracted from testis of wild type, cpes mutant, and various rescues expressed in germ cells using vasa-Gal4. In contrast to Figs 1 and 2, here we used whole male organ (testis, accessory glands, collecting ducts, and ejaculatory bulb) for lipid extraction. (H) The amounts of CPE and its subspecies in wild type, cpes mutant, and various rescues are shown. (I) Ceramide and its subspecies are shown in picomoles per 100 μg of carbon. (J) Hexosylceramide subspecies amounts are shown in bar diagram. (K) Amount of SM and its subspecies are shown as picomoles per 100 μg of carbon. Fluorescent blue green part of the bar represents SM species with net 2 double bonds in them. (L) TLC of lipids extracted from 50 pairs of testes from each genotype. The position of SM on TLC is indicated by an arrow. Original uncropped TLC image can be found in S1 Raw Images. DHSPH, SPH, SPD SFA, MUFA and PUFA. Statistical significance was calculated using mean, SD, and N in Prism 8. The 2-way ANOVA multiple comparison was used to calculate p-values where **** p ≤ 0.0001, *** p ≤ 0.001, ** p ≤ 0.01, * p ≤ 0.05, and ns p >0.05. Three independent biological replica [file pbio.3001599.s005.pdf]

Figure S6  
Kunduri et al., 2022

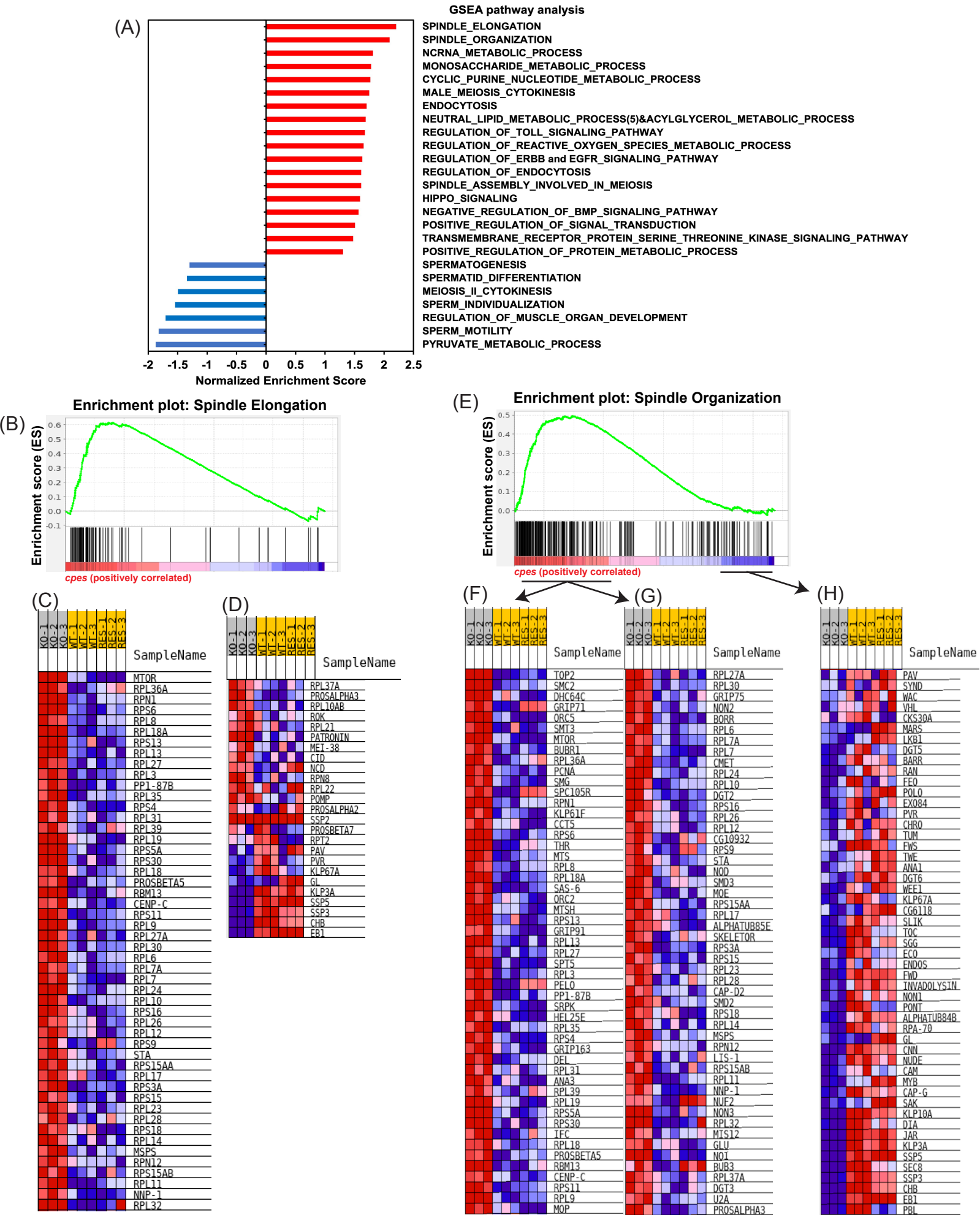

Supplement: S6 Fig — (A) GSEA pathway analysis of RNA seq data from w1118, cpes mutant, and bam-Gal4>UAS-CPES rescue testis. Three independent biological replicates were taken for each sample and RNA was extracted from 200 pairs of testes for each biological replicate. (B) Enrichment plot showing genes involved in spindle elongation are positively correlated with cpes mutants compared to w1118 and bam-Gal4>UAS-CPES rescue. (C and D) Heatmap shows spindle elongation genes are enriched in cpes mutants. (E) Enrichment plot showing genes involved in spindle organization are positively correlated with cpes mutants compared to w1118 and rescue. (F and G) Heatmaps show top 100 enriched genes in cpes mutants. (H) Heatmap shows the top 50 genes enriched in wild type and rescue. The data underlying the graphs shown in this figure can be found in S2 Data. (PDF) [file pbio.3001599.s006.pdf]

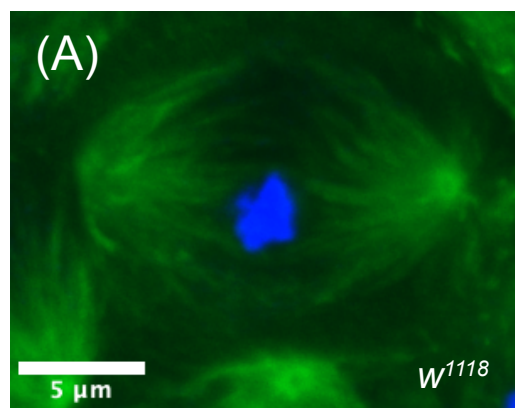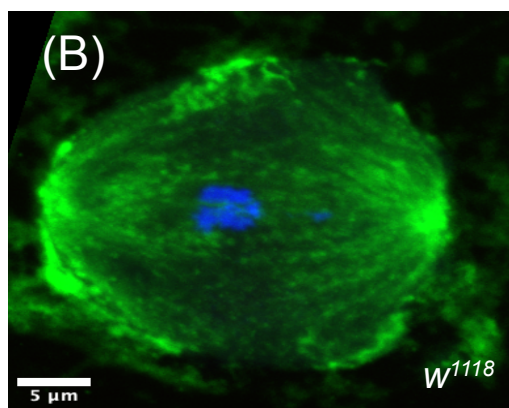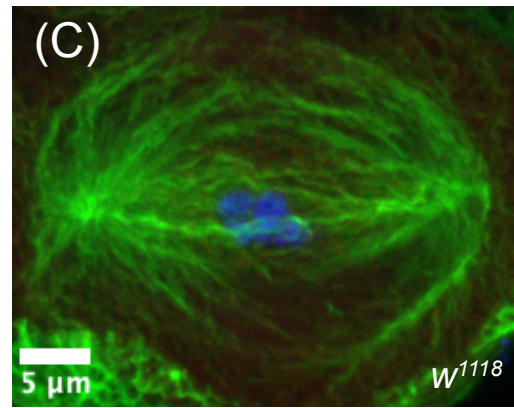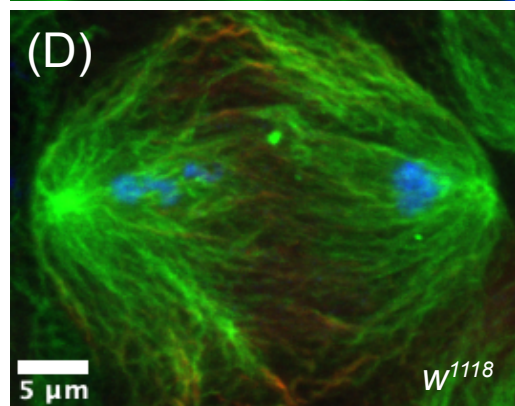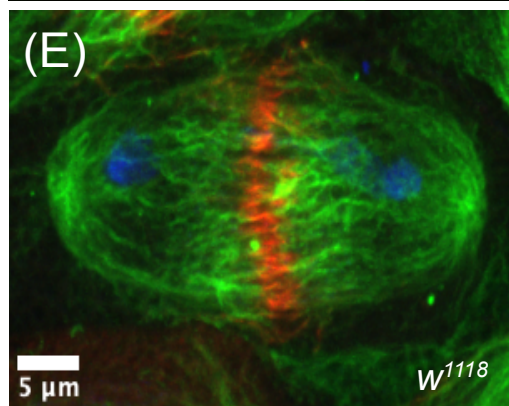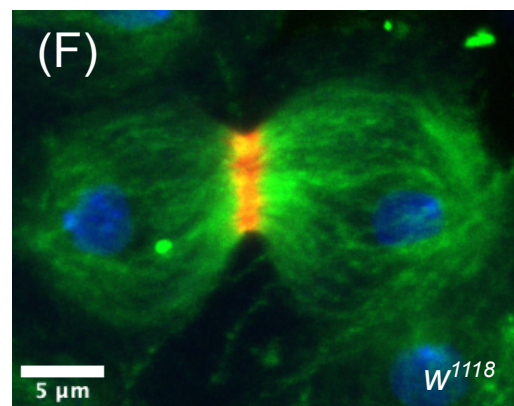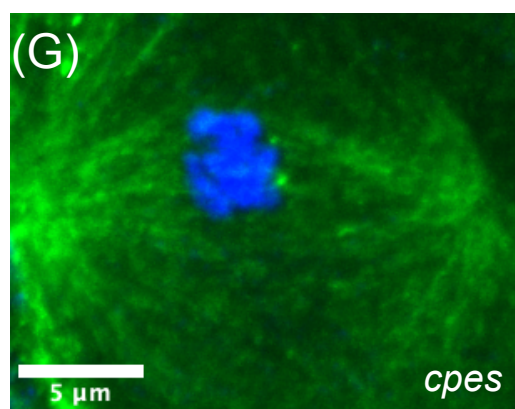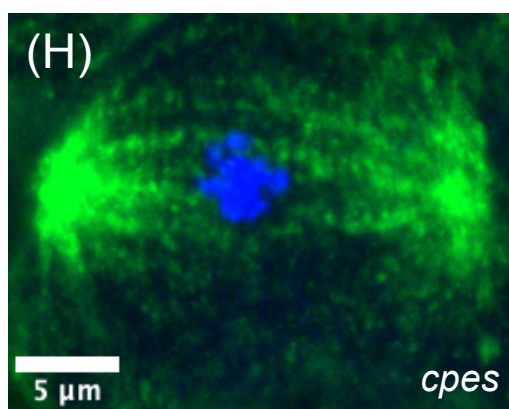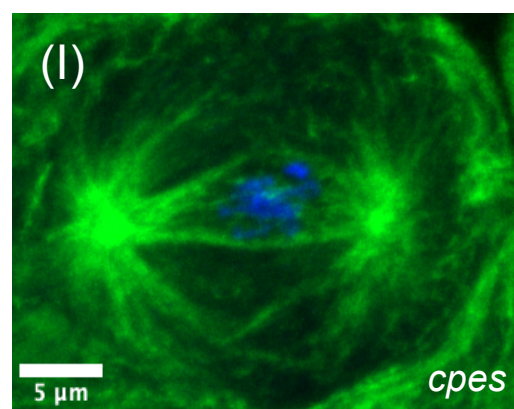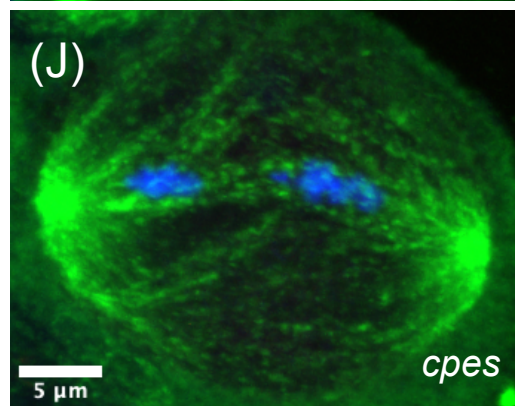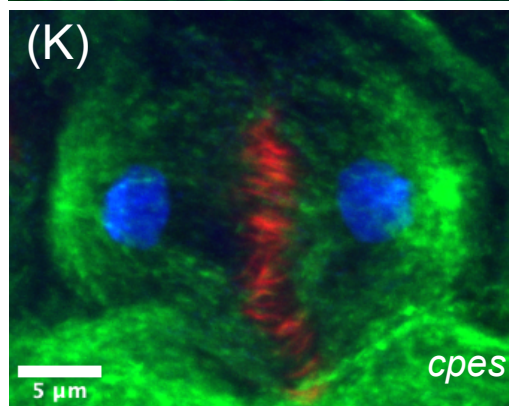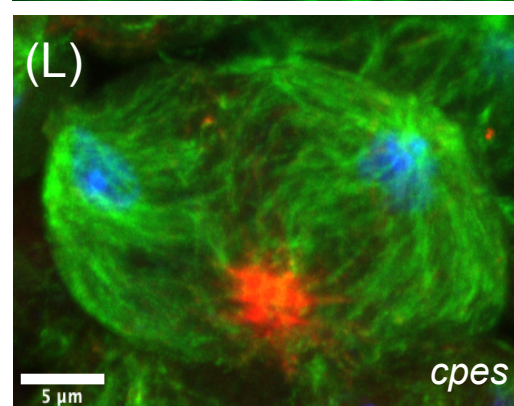

Supplement: S7 Fig — Microtubules are immunostained with alpha tubulin antibody and chromosomal DNA with DAPI. Confocal images of wild-type spermatocytes in metaphase (A–C), anaphase (D), early telophase (E), and late telophase (F). Confocal images of cpes mutant spermatocytes in metaphase (G–I), anaphase (J), early telophase (K), and late telophase (L). (PDF) [file pbio.3001599.s007.pdf]

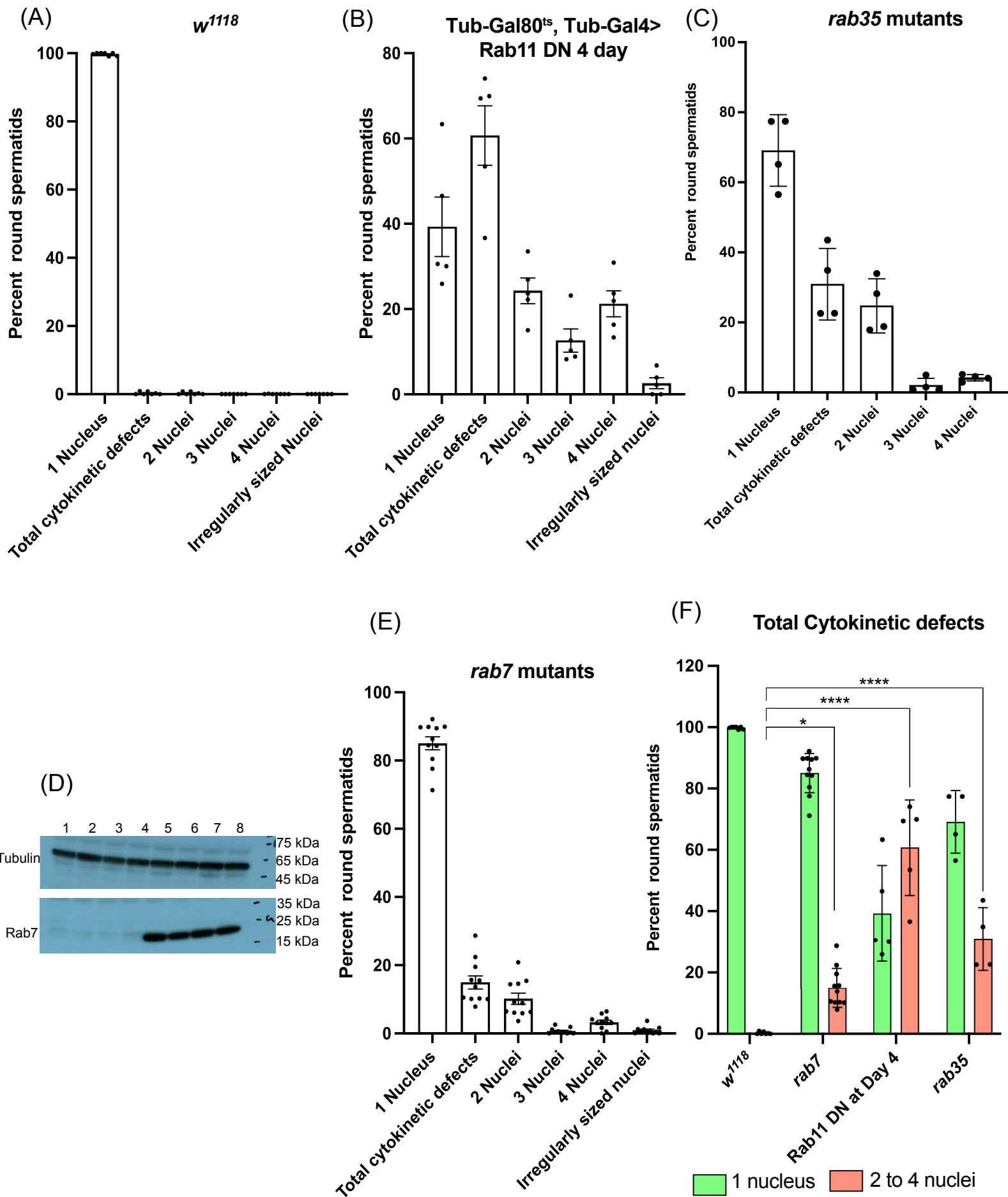

Supplement: S8 Fig — (A) Measurement of cytokinetic defects in testes squash preparations of wild-type samples, 7 independent experiments, and total round spermatid count of about (n = 6,000). Fraction of round spermatids showing nucleus to mitochondria ratio 2:1 (2 nucleus), 3:1 (3 nucleus), 4:1 (4 nucleus), and 4 irregularly sized nucleus with 1 mitochondrion are depicted. (B) Cytokinesis defects in Rab11 dominant negative expressing gonads at fourth day, 5 independent experiments, and total round spermatid count of about (n = 1,500). (C) Cytokinetic defects in Rab35 null mutants, testis from 1–2-day-old adult males were used for analysis, 4 independent experiments, and a total of round spermatid count equals to 1,250. (D) Western blotting of protein extracts from control and Rab7 mutants using Rab7 and tubulin (loading control) antibodies. Lanes 1–4 (Rab7 mutants) and lanes 5–8 (Control). Original uncropped western blot image can be found in S1 Raw Images. (E) Fraction of cytokinetic defects in Rab7 null mutant pupal gonads (24–48 h post pupation), 11 independent experiments, and round spermatid count of about (n = 1,100). (F) Comparison of total cytokinetic defects between Rab7, Rab35 mutants, and Rab11DN (sum of all forms of cytokinetic defects including nuclear to mitochondria ratios 2:1, 3:1, 4:1, and irregularly sized nucleus). Each dot in each graph represents an independent experiment (n) that consisted of 10 individual testis/gonads. The data underlying the graphs shown in this figure can be found in S2 Data. (PDF) [file pbio.3001599.s008.pdf]

**Figure S9**  
**Kunduri et al., 2022**

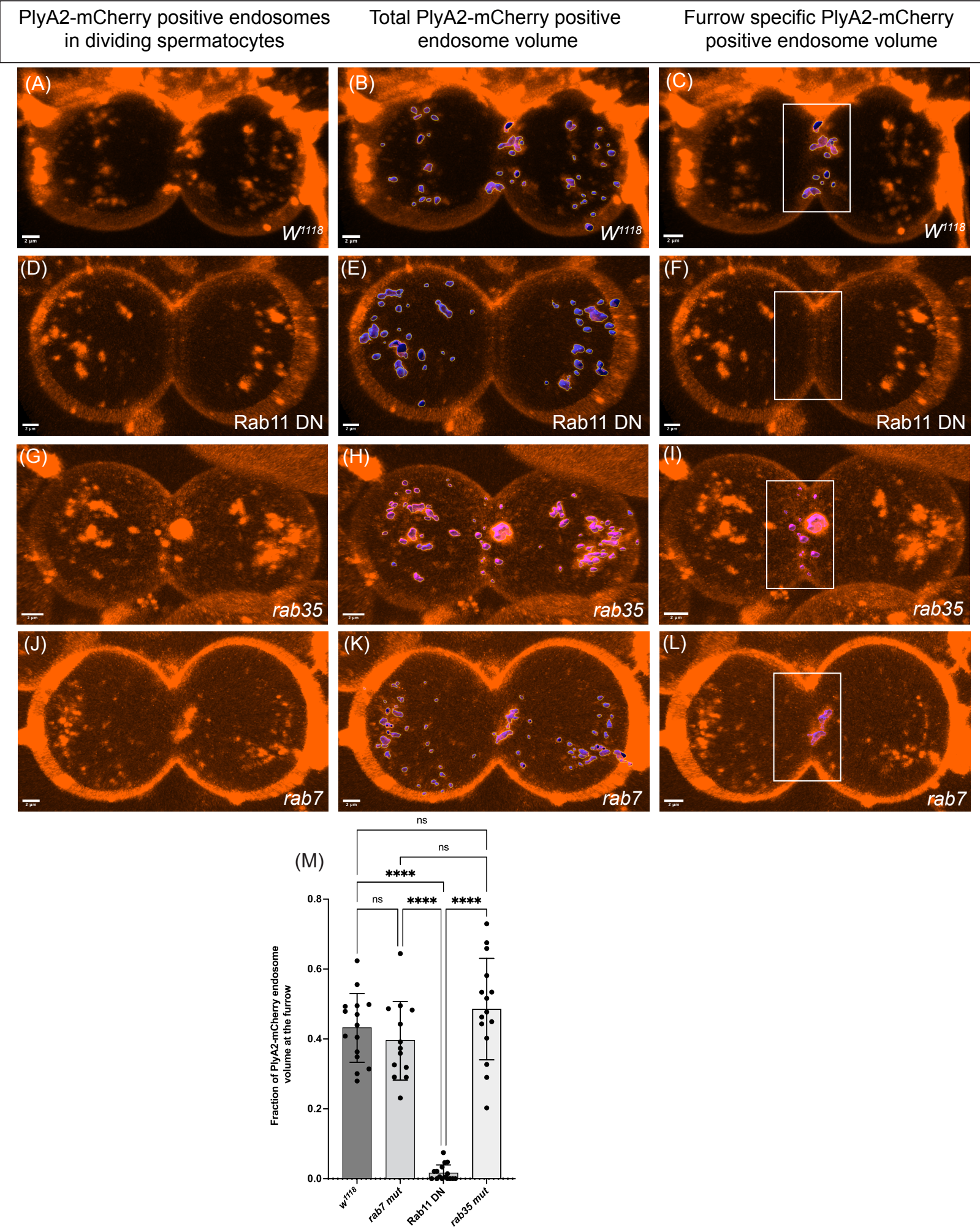

Supplement: S9 Fig — Spermatocytes from all backgrounds were treated with PlyA2-mCherry and imaged live to capture cytokinetic step. Endosome surfaces in 3D images were traced using surfaces tool in Imaris software by setting up appropriate thresholds. Images B, E, H, and K show the total endosomal surfaces traced using surfaces. Note that the plasma membrane and other areas of the images were not considered for calculating endosomal volume. Images C, F, I, and L show furrow-specific endosomal volume where white rectangle box indicates area chosen for measuring furrow-specific volume. (A–C) w1118 spermatocytes, (D–F) Rab11 DN expressing spermatocytes, (G–I) Rab35 mutant spermatocytes, (J–L) Rab7 mutant spermatocytes. (M) Fraction of endosomes enriched at the furrow was calculated for all the backgrounds by dividing furrow-specific endosomal volume with total endosomal volume. Each dot in the graph represents an independent spermatocyte undergoing division from 3 independent experiments. w1118 (n = 15), rab7 mutant (n = 13), Rab11 DN (n = 16), and rab35 mutant (n = 15). Statistics were performed using ordinary 1-way ANOVA Turkey’s multiple comparisons in Prism9, ns = p > 0.05 and **** = p < 0.0001. The data underlying the graphs shown in this figure can be found in S2 Data. (PDF) [file pbio.3001599.s009.pdf]

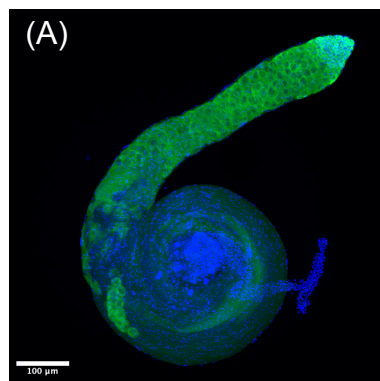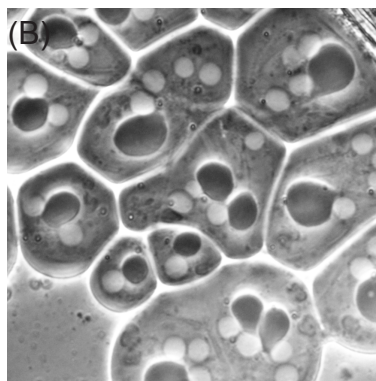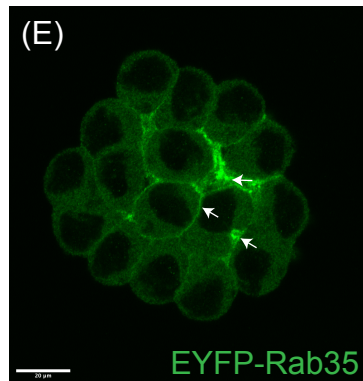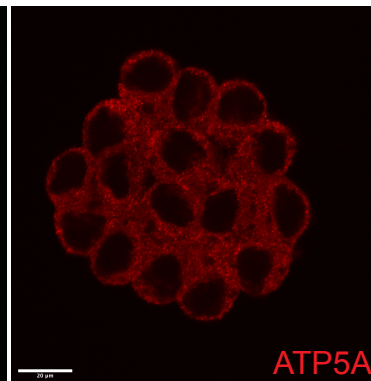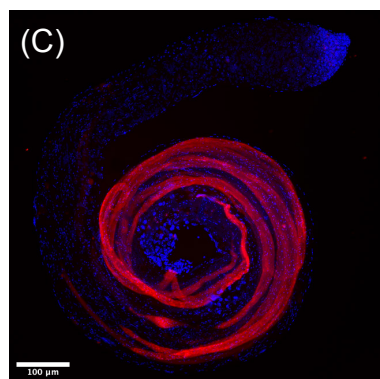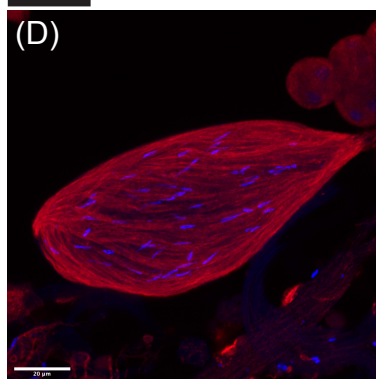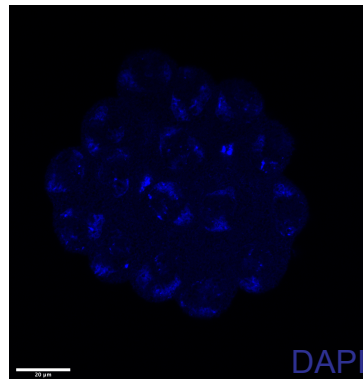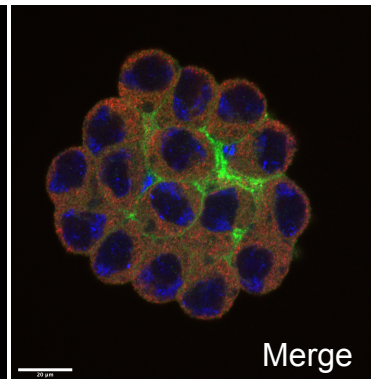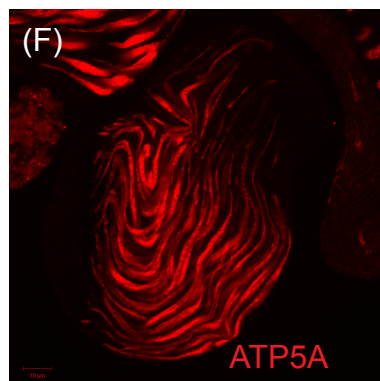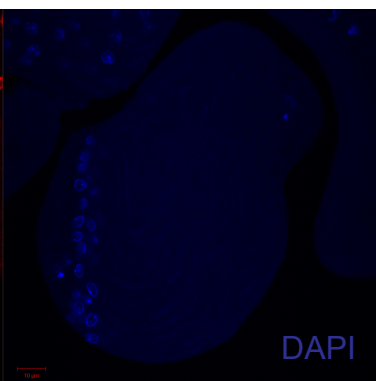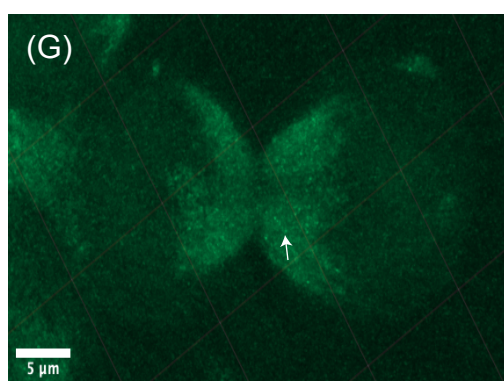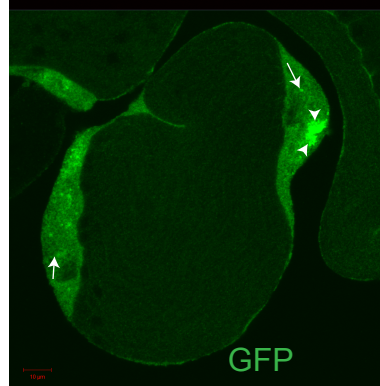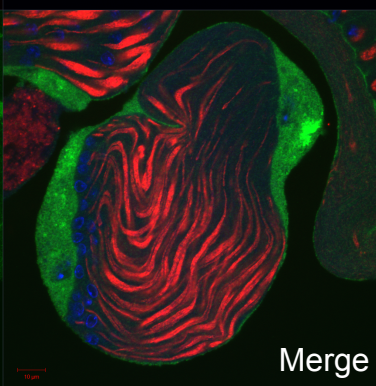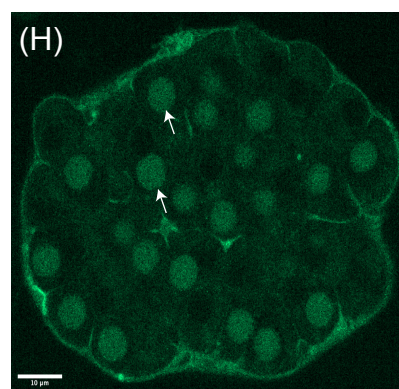

Supplement: S10 Fig — (A) Confocal image showing germ cells in rab 35 mutant testis that are immunostained with Vasa antibody (green) and DNA (blue). (B) Phase contrast image showing round spermatids in testis squash preparations of rab35 mutant. (C) Confocal image showing elongated spermatids of rab35 mutant testis immunostained with cleaved caspase (DCP1) (red) and DNA (blue). (D) Confocal image showing early elongated spermatids in rab35 mutant testis squash preparations immunostained with alpha tubulin antibody (red) and DNA (blue). (E) Confocal images showing spermatocyte cysts that are expressing EYFP-Rab35 (endogenous promoter). Testis squash preparations of EYFP-Rab35 were immunostained with GFP antibody (green), ATP5A antibody to detect mitochondria (red), and DNA (blue). Cyst cell and its membranes are shown with arrow. (F) Confocal image showing early elongating spermatids in samples from E, cyst cells were shown with arrow and endosomes with arrow heads. (PDF) [file pbio.3001599.s010.pdf]

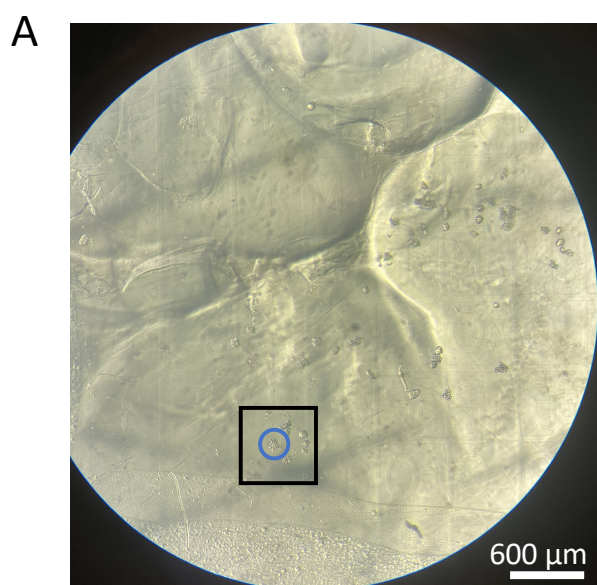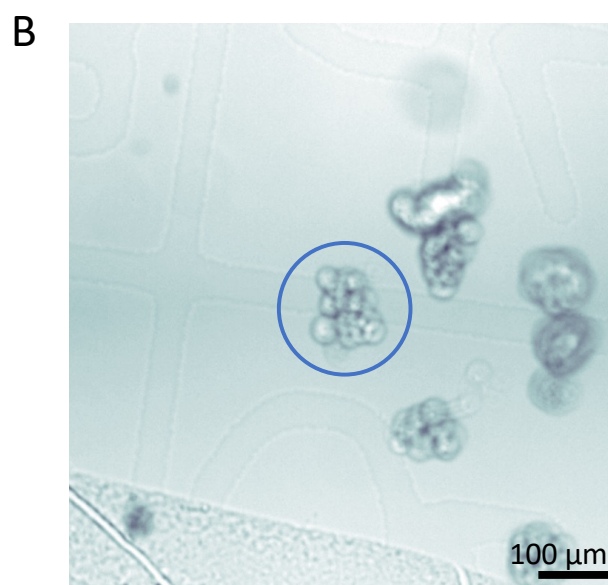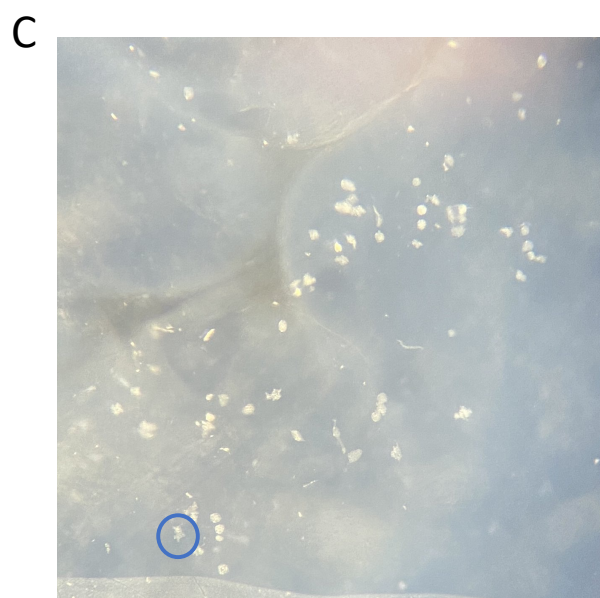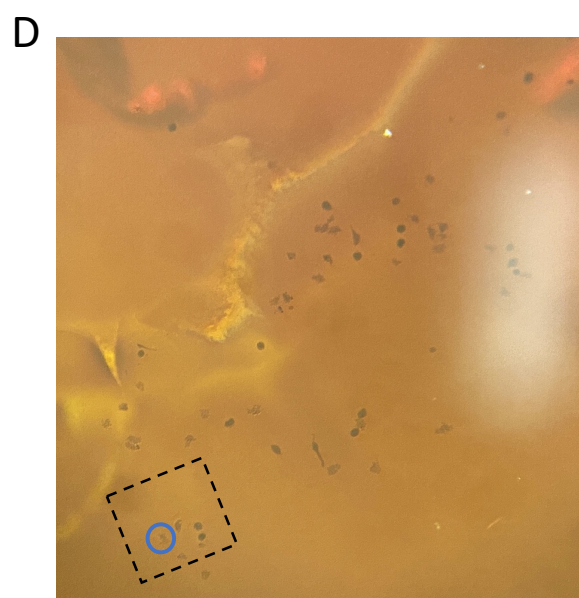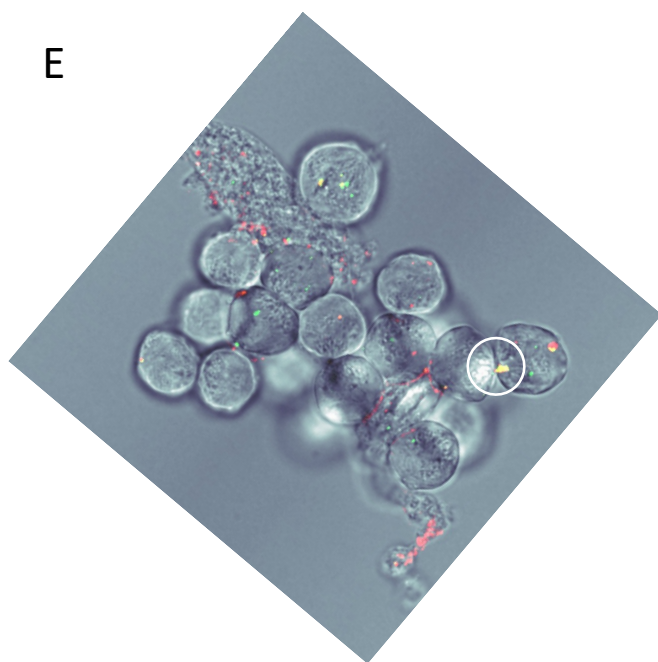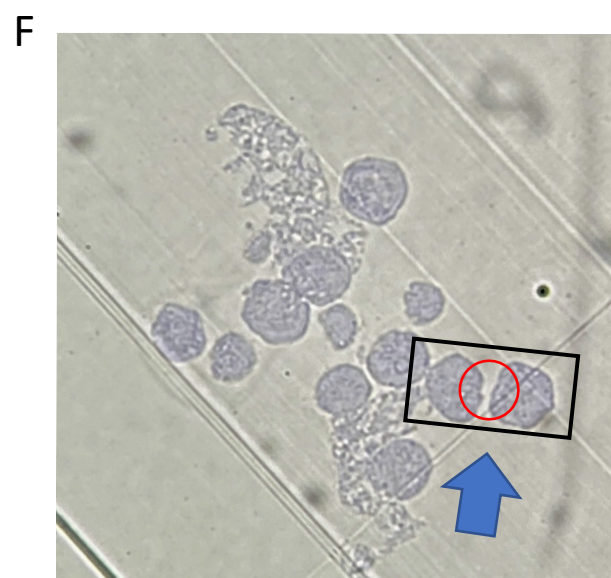

Supplement: S11 Fig — (A) Drosophila cysts were dissected onto a gridded cover slip, after fixation and embedded in 4% agarose. The cyst of interest is circled and is the same as the cyst shown in Fig 7A. (B) Higher magnification image of boxed area in (A). (C) Light microscopy overview of full field before EM processing. (D) Same field after EM processing. The same pattern of dissected cysts is visible, suggesting spatial retention of sample. Trimmed area approximated with a dashed rectangle. (E) Field of view (orthogonal to Fig 7A) with doubly labeled cleavage furrow of interest circled. (F) Sectioning at the microtome proceeded until furrow of interest was almost exposed (red circle). FIB-SEM imaging was executed in the volume indicated by rectangle, in direction of arrow. (PDF) [file pbio.3001599.s011.pdf]
