# Supplementary material for: Delivery of ceramide phosphoethanolamine lipids to the cleavage furrow through the endocytic pathway is essential for male meiotic cytokinesis
Source: PLoS Biol. 2022 Sep 28;20(9):e3001599. doi: 10.1371/journal.pbio.3001599 (PMC9550178; doi:10.1371/journal.pbio.3001599)

Raw western blot data used in Fig.S8D

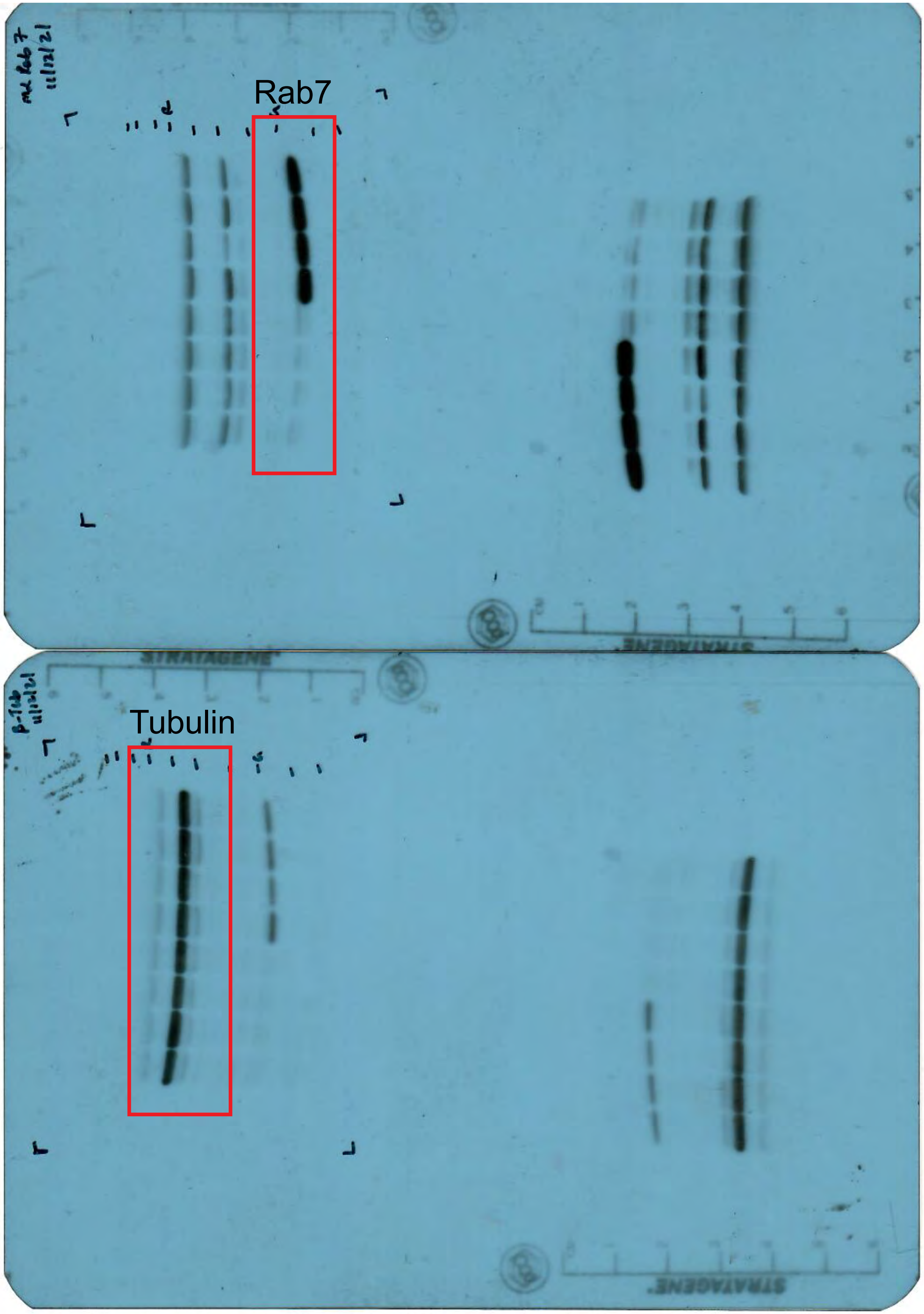

Raw thin layer chromatography data used in **Fig.S5L**

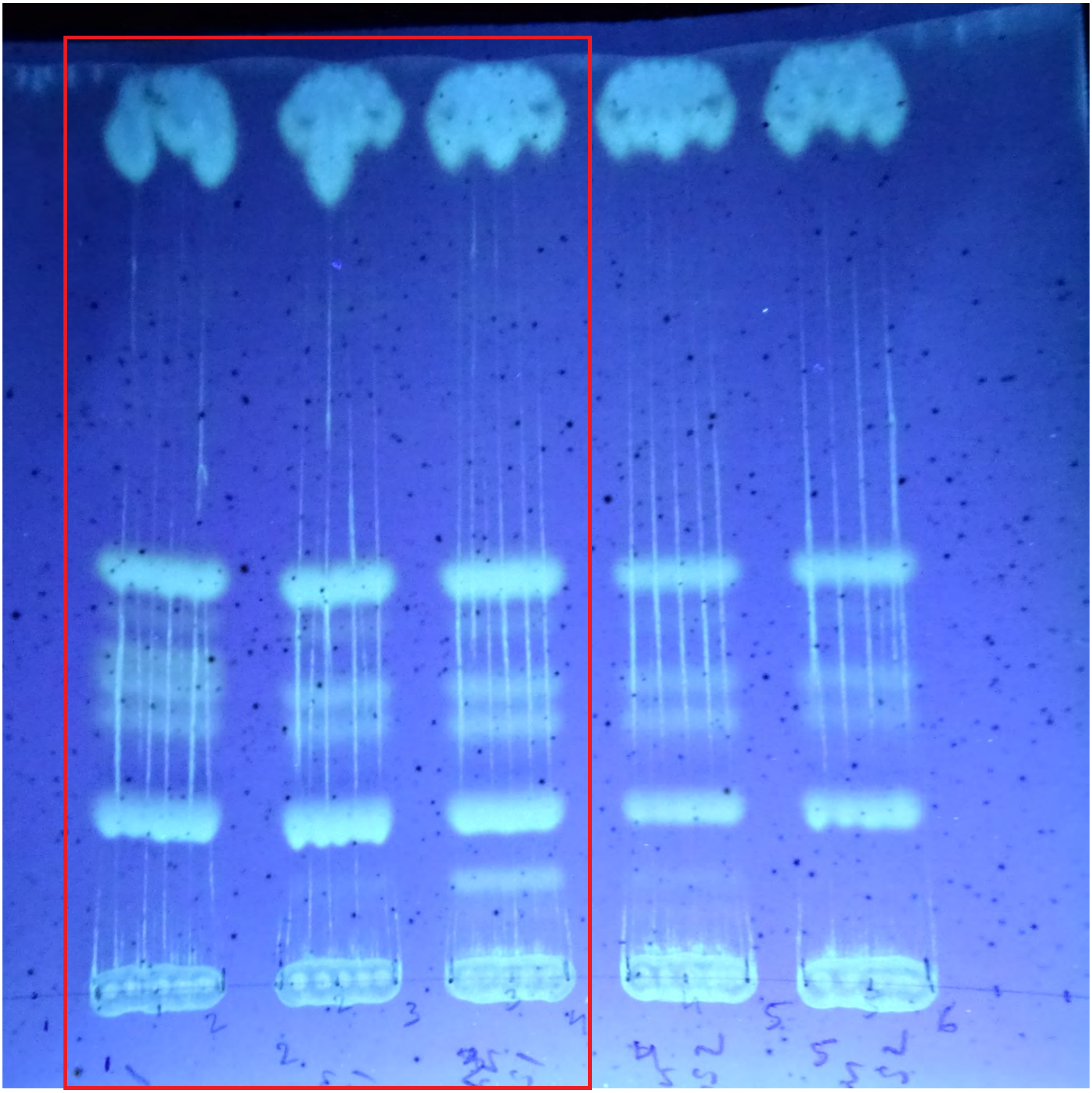

Supplement: S1 Raw Images — Represented blot in the figure was shown in red box. (PDF) [file pbio.3001599.s033.pdf]
