# Supplementary material for: Delivery of ceramide phosphoethanolamine lipids to the cleavage furrow through the endocytic pathway is essential for male meiotic cytokinesis
Source: PLoS Biol. 2022 Sep 28;20(9):e3001599. doi: 10.1371/journal.pbio.3001599 (PMC9550178; doi:10.1371/journal.pbio.3001599)
Supplement: S1 Table — (DOCX) [file pbio.3001599.s037.docx]

| Name | Sequence (5’-3’) | Description |
| --- | --- | --- |
| Aedes FW | ATAgAATTCAAAATggTggggCCCAgTTCgCAAATC | To PCR amplify Aedes aegypti CPES ORF and subclone into PUAST vector; EcoRI site is underlined. |
| Aedes Rev | CCCTCTAgATTACgTAgAATCgAgACCgAggAgAgggTTAgggATAggCTTACCACCCAACgATgTATTgTTACCAgT | To PCR amplify Aedes aegypti CPES ORF with V5 tag at C-terminus and subclone into PUAST vector; XbaI site is underlined. |
| B mori FW | ATAgAATTCAAAATgTggCCACCATCgCAAgCCAgC | To PCR amplify Bombyx mori CPES ORF and subclone into PUAST vector; EcoRI site is underlined. |
| B mori Rev | CCCTCTAgATTACgTAgAATCgAgACCgAggAgAgggTTAgggATAggCTTACCACCTCTACCATCAACggAggTTTTAAC | To PCR amplify Bombyx mori CPES with V5 tag at C-terminus and subclone into PUAST vector; XbaI site is underlined. |
| CPES active site FW | CCTCCggCTACTACgTAgCCggCCTgTgCgCCggACTCggCTgC | To replace active site Aspertates (D214A and D218A) with Alanine using site directed mutagenesis method. |
| CPES active site Rev | gCAgCCgAgTCCggCgCACAggCCggCTACgTAgTAgCCggAgg | To replace active site Aspertates with Alanine (D214A and D218A) using site directed mutagenesis method. |
| CPES FW | ACAAgATCTATgATCggACCCAgTTCgCAg | To PCR amplify CPES ORF with active site mutation and V5 tag at the C-terminus and subclone into pUAST vector. BglII site is underlined. |
| CPES V5 Rev | ACACTCgAgTCACgTAgAATCgAgACCgAg | To PCR amplify CPES ORF with active site mutation and V5 tag at the C-terminus and subclone into pUAST vector. XhoI site is underlined. |
| PlyA2Fw | CCCCATATggCCTACgCCCAgTgggTC | To PCR amplify PlyA2 ORF and subclone into pET24a vector for protein expression and purification. NdeI site is underlined. |
| mCherry Rev | CCCCTCgAgCTTgTACAgCTCgTCCATg | To PCR amplify PlyA2-mCherry and subclone into pET24a vector for protein expression and purification. XhoI site is underlined. |

Table S1
