# Supplementary material for: Delivery of ceramide phosphoethanolamine lipids to the cleavage furrow through the endocytic pathway is essential for male meiotic cytokinesis
Source: PLoS Biol. 2022 Sep 28;20(9):e3001599. doi: 10.1371/journal.pbio.3001599 (PMC9550178; doi:10.1371/journal.pbio.3001599)
Supplement: Attachment — Submitted filename: Response to reviewers.pdf [file pbio.3001599.s038.pdf]

Response to Reviewers

We thank all four reviewers for comments and suggestions that have improved the quality of the manuscript considerably. We hope the changes we have incorporated following their suggestions and the clarifications we have provided in our response to the reviewers would provide sufficient clarity and elevate the quality of the work for publication in PLOS Biology. We have performed correlative light and electron microscopy-focused ion-beam-scanning electron microscopy (CLEM-FIB-SEM) and correlative light and electron microscopy-scanning electron microscopy-transmission electron microscopy (CLEM-SEM-TEM) to visualize the CPE containing endosomal structures. We show that these are multivesicular bodies and they deliver intraluminal vesicles to the cleavage furrow. These new findings raise interesting possibilities about topology, organization, and orientation of lipids/proteins delivered by these vesicles. Future endeavors should enable us to understand how these lipids are incorporated into expanding membranes of the furrow.

Further, we have reanalyzed the RNAseq data and presented it more in alignment of the observed phenotypes and have removed sentences that allude to hopes of mechanistic insight. We have validated the expression pattern in stage specific Gal4/PUAST system used in our study for expression in male germ line. Additionally, we have provided comparison of pUAST and pUASP reporters and provided the data. We have also examined cytokinesis defects in a null mutant of Rab7 and flies expressing dominant negative Rab11 allele.

We appreciate the reviewers' comments and have included new experiments and a point-by-point response to each of the reviewers' concerns. We feel that the reviewers' comments have strengthened the manuscript and hope that it is now suitable for publication in PLOS Biology. We thank you for your insight and invaluable suggestions.

While addressing the concerns of the reviewers we have made extensive changes to the organization of the manuscript including several changes to the figures. The main edits are:

1. The title of manuscript has now been changed as advised by reviewer 1. It now reads as "**Endosomes deliver ceramide phosphoethanolamine with unique acyl chain anchors to the cleavage furrow during male meiotic cytokinesis**".
2. Figure 1A, a brief flow chart of *de novo* biosynthesis of sphingolipids has now been added to Figure 1.

3. Figure 2M: Statistical analyses of cytokinetic defects in wild type, mutant, and rescue round spermatids.
4. Figure 3N: Quantification of cytokinetic defects observed in various testes, rescued using germline stage-specific Gal4 drivers.
5. Figure S5: Old Figure 4 has been edited and now moved to Figure S5 as suggested by reviewer 4.
6. Figure 4: This is reorganized from old Figure 5. Previous Figure 5A-C and Data S3 and S4 has been re-organized and presented as Figure S6. New Figure 4A, C and D are panels from previous Figure 5D-F. Panel 4B is a new panel that includes statistical analysis of spindle organization defects in wild type, mutant and rescue spermatocytes undergoing meiosis.
7. New Figure 5 is the old Figure 6. Panel Q is a new panel with statistical analysis of colocalization of various Rabs with endocytosed CPE binding protein PlyA2.
8. New Figure 6 is old Figure 7.
9. Figure 7: This is a new Figure. The figures describe experiments done using CLEM-FIB-SEM data of endocytosed Rab7/PlyA2 vesicles and Anillin-mRFP/Rab7-EYFP positive vesicles.
10. Figure 8: This is a new Figure of CLEM-SEM-TEM of endocytosed Rab7 vesicles at the cleavage furrow.
11. Figure S3: New Figure - analysis of RNAseq based on stage specific genes.
12. Figure S4: New Figure - analysis of stage specific expression of male germ line lineage drivers and comparison of pUAST and pUASP reporters.
13. Figure S7: New Figure - analyses of Rab7 null and dominant negative Rab11 mutants for defects in male meiosis
14. Figure S8: New Figure - Supporting data for registration of light microscopy images with CLEM-FIB-SEM and CLEM-TEM experiments described in Figures 7 and 8.
15. Movie S1: New movie - Mitotically dividing control and *cpes* mutant larval neuroblasts. Other movies have been accordingly relabeled.
16. Movie S13: New Movie - Endocytosis of PlyA2 containing endosomes in Rab7 mutant pupal male spermatocytes.
17. Movie S14: New Movie - FIB-SEM sections of male spermatocytes undergoing meiosis

1 with Rab7-PlyA2 double positive endosome at the furrow.

18. Movie S15: New Movie - Colocalization of Anillin-mRFP and Rab7-EYFP during meiosis 1 (used for CLEM-FIB-SEM and CLEM-SEM-TEM).
19. Movie S16: New Movie - Volumetric rendering of the FIB-SEM data shown in new Figure 7.
20. Movie S17: New Movies – Volumetric rendering of Rab7 vesicles docked to the ingressing membrane of the furrow.
21. Movie S18: New Movie - Colocalization of Anillin-mRFP and Rab7-EYFP during meiosis 1 in cpes mutant spermatocytes.
22. We have incorporated all other editorial and other corrections suggested by all four reviewers.

Reviewer 1:

OVERVIEW

Successful cytokinesis requires the proper composition of membranes at the cleavage furrow. Among the lipids shown to be important for this process are sphingolipids, yet their regulation and contribution are poorly understood. Here, the authors examine the role of ceramide phosphoethanolamine (CPE), the insect structural analog of sphingomyelin (SM), in Drosophila spermatocyte cytokinesis. They first perform lipidomic analysis on heads and gonads of wild-type flies and determine that CPE levels are dramatically elevated in testes vs. ovaries and male and female heads. Ceramide levels are also elevated in testes, whereas hexosylceramide (HexCer) is elevated in heads from both males and females. Upon further examination, they determine that testes are enriched for CPEs containing very long chain fatty acids (VLCFAs; C18-C24), consisting of either sphingosine linked to mono-unsaturated FAs (SPH-MUFA) or sphingadiene linked to saturated FAs (SPH-SFA). To test the function of CPEs in testes, they examine mutants for CPE synthase (cpes), which they had previously shown is required for male

fertility and sperm production. They identify spermatocyte cytokinesis, spermatid polarity and individualization defects in cpes mutant male germ cells and show that these phenotypes are rescued by ubiquitous expression of CPES. Moreover, cpes mutant testes lack CPE and contain excess ceramide, phenotypes that are also reversed by CPES expression. Importantly, they demonstrate that germline expression of CPES in spermatogonial cells using bam-Gal4, but not other germline or cyst cell drivers, fully rescues the mutant phenotypes and restores male fertility. They demonstrate that V5-tagged CPES colocalizes with the Golgi protein GM130, consistent with its known role in CPE synthesis in the Golgi. They further show that expression of CPES from other species (Aedes and Bombyx, but not T. urticae) rescues sperm development in cpes mutants. However, expression of human SM synthase (hSMS1) fails to rescue sperm development, which was surprising given that hSMS1 rescued glial defects they had previously reported in cpes mutants. This indicates that SM is not sufficient to replace CPE during sperm development. The authors perform RNAseq on wild-type, cpes mutant and rescued testes. They find that transcripts involved in spindle elongation, spindle organization, male meiosis and other processes are more abundant in cpes mutants relative to wild-type and rescued testes. They examine cpes mutants for defects in spindle microtubules and in cytokinesis and discover defects in central spindle formation and constriction, but not in myosin regulatory light chain (Sqh), PIP2 (PLCdelta-PH) or PIP3 (tGPH) distribution at the cleavage furrow. These defects are rescued by CPES expression. To examine CPE in cells, they use exogenously applied PlyA2-mCherry as a marker and show that this binds the plasma membrane (PM) in wild-type but not cpes mutant spermatocytes, confirming absence of CPE in the outer leaflet of the PM. They also examine endocytoses PlyA2-mCherry and find that it colocalizes with the endosomal Rab4, Rab7 and Rab11, but not with Golgi-localized Rab6. They demonstrate colocalization of PlyA2-

mCherry with Rab7 and Rab11-positive endosomes at the midzone, suggesting that CPE is transported on endosomes to the midzone during cytokinesis.

Overall, the results provide a significant advancement in our understanding of Drosophila sphingolipids, as well as a novel in vivo model to study the role of CPE in cytokinesis. Nonetheless, I feel there are significant deficits in the rationale and interpretation of two of the experiments that must be corrected prior to publication. These, as well as a number of general and specific comments on the manuscript, are listed below.

MAJOR COMMENTS

1. The rationale for using RNAseq to "determine the mechanism by which CPES regulates meiotic cytokinesis" is unclear. The results of the RNAseq experiments can be trivially explained by a dearth of later stages of spermatogenesis, resulting in an enrichment of transcripts involved in male meiosis and reduced levels of transcripts involved in meiosis II cytokinesis and spermatid differentiation. Consistent with this, cpes mutant testes appear to have unusually large numbers of spermatocytes (Fig. 2B) and few elongated spermatid cysts, especially at late stages (Fig. 2K), compared to wild-type and rescued testes. Thus, the argument for using these results as a reason for examining spindle behavior is rather weak. Moreover, the transcripts of at least seven genes known to be required for cytokinesis (dia, fwd, fws, onr, pav, pbl, sec8), and in two cases (onr, sec8) spermatid polarity, are reduced in cpes mutants. It is unclear why the localization of at least some of the encoded proteins was not examined as part of the analysis of the cpes mutant phenotype, as defects in the abundance, distribution or function of these gene products could potentially explain the observed cytokinesis defects. This paragraph should be rewritten to highlight information that actually can be gleaned from the analysis (differential

distribution of stages of sperm development, increase/decrease in particular pathways in mutant vs. controls) rather than as a way to figure out the mechanism.

We thank the reviewer for the comments. In the revised version of the manuscript, the RNA seq data has been moved to the supplemental section. As suggested by the reviewer, we have reanalyzed the data from RNAseq experiments. The data was analyzed for the top 50 genes that were enriched in individual cell types as described by Witt. D et al (eLife 2019;8: e47138 DOI:10.7554/ eLife.47138). The analysis indicated that gene expression largely complemented the phenotypes obtained from imaging experiments i.e., there was a general enrichment for genes expressed in the earlier stages and a downregulation of genes expressed in the post-meiotic stages of spermatogenesis (Fig. S3). The narrative has been appropriately edited to indicate that analysis of RNAseq data is supportive of the observed phenotypes broadly. We have avoided sentences implying that RNAseq analysis was performed to extract mechanistic insight.

Since we were unable to obtain the antibodies for the proteins suggested by the reviewer, we tried localization of ubiquitously expressing pav-GFP and pbl-GFP transgenic flies in control and *cpes* mutant background. Pav-GFP showed central spindle localization like mcherry-Feo, (Reviewer's perusal only Movie 1) and pbl-GFP localizes to the spindle and is expressed very weakly in the transgenic flies (Reviewer's perusal only Move 2). The comparisons from studying the localization and dynamics from the control and mutant flies did not provide any additional information than that was obtained using mcherry-Feo which provided stronger signal and better resolution of central spindle dynamics (Fig.4, Movie S2 - Movie S10).

<u>Revised manuscript (lines 314-339)</u>

Transcriptomic and genetic dissection of spatiotemporal role of CPES in spermatogenesis

Spermatogenesis is a highly conserved process across various taxa that is facilitated by highly dynamic transcriptome. An earlier study leveraged single cell RNAseq and unsupervised clustering to identify all the major cell classes of the sperm lineage and validated them with previously studied marker genes [33]. To characterize cell type specific transcriptional signatures in *cpes* mutants *in vivo*, we performed total RNA-seq with dissected testis from w^{1118}, *cpes* mutant and *bam*-Gal4>UAS CPES rescue. Using Gene Set Enrichment analysis (GSEA) we compared our RNAseq results with the top 50 expressed genes from each cell type/cellular status that were previously classified to confirm the presence of germline stem cells (GSC), spermatogonia, spermatocytes, and spermatids (germ cells) as well as cyst stem cells, terminal epithelial cells and hub cells (somatic cells) [33]. We found that gene sets corresponding to GSC, early spermatogonia and late spermatogonial cells are enriched in *cpes* mutants compared to wild type and rescue testis (Fig.S3). In contrast, gene sets corresponding to early and later spermatocytes, early, and mature spermatids are enriched in wild type and rescue testis compared to *cpes* mutant testis. These results suggested that CPES might play important roles during late spermatogonial, early and late spermatocyte stages for successful completion of spermatogenesis.

Fig.S3. Analysis of cell type specific transcriptomic signatures in cpes mutants: *(A) GSEA using top 50 cell type specific expressed gene sets and compared between cpes versus wild type (WT) and bam-Gal4>UAS CPES rescue (Rescue). Red bars indicate gene sets enriched in cpes mutants and blue bars indicate gene sets enriched in WT & Rescue. (B) enrichment plots for each of the cell type shows the distribution of individual genes in each set (vertical lines on red to blue parallel bar). Genes with positive enrichment score (ES) indicate that they are enriched in cpes mutants whereas negative enrichment score indicate that they are enriched in WT and Rescue. (C) Heatmap comparision of cell type specific enriched gene sets in w wild type (W1118), cpes and*

Rescue (bam-Gal4>UAS CPES rescue). Red indicates gene sets enriched in cpes mutants and blue indicate gene sets enriched in WT & Rescue.

2. In a related point, it was not immediately obvious what Data S3 and S4 correspond to nor how to interpret the results (for example, why are some of the same genes shown in both?). These data files should either be described in the text or a description should be included in the files. Also, the figure in Data S3 is impossible to print. It would help to break this into multiple panels that will fit on 1-2 pages rather than including the whole dataset on a single page. In addition, the assembly of the figure in Data S4 resulted in some of the text being pixelated and unreadable, including the last gene listed. It is unclear why this figure had to be assembled in multiple sections, which raises questions about how the data in this figure were selected. This should be explained in the text.

We apologize for not making the point clear. The manuscript has been experimentally revised and additional figures have been added. In the interest of space and significance of the data, we have now moved all the RNA seq analysis to the supplemental section. In the revised manuscript, Figures 5A, B and C and Data set S3 and S4 have been reorganized into Figure S6. The analyses indicated that the genes categorized under 'spindle organization' and 'spindle elongation' positively correlated with the *cpes* mutants. The two sets have significant overlap because several of the genes implicated in spindle organization have also been categorized under spindle elongation and hence the duplication of genes in the two categories. As suggested by the reviewer, we have reorganized this data in Figure S6.

Revised manuscript (lines 503-531)

Aberrant central spindle behavior but not localization of PIPs at the plasma membrane is responsible for defective meiotic cytokinesis in *cpes* mutants

Precise organization of central spindle microtubule is not only required for initial cleavage furrow formation but also for maintenance of contractile structures during furrow ingression. Several microtubule interacting proteins including Fascetto, kinesin 6 family member MKLP1/Pavarotti (pav), chromokinesin klp3A, Orbit etc., are enriched in the central spindle midzone and are essential for cytokinesis [39]. To determine the transcriptional signatures relating to spindle organization and function in our RNAseq data, we performed GSEA pathway comparisons between w^{1118} (WT), *cpes* (KO) and rescue (RES) samples. As shown in Fig.S6A. we found several pathways including spindle organization, metabolism, endocytosis, signaling, male meiotic cytokinesis, spermatid differentiation, sperm individualization etc., are significantly altered in *cpes* mutants (Fig. S6A). Red bars in the Fig. S6A indicate gene sets enriched in *cpes* mutants and blue bars indicate gene sets enriched WT & RES. GSEA showed many genes involved in spindle elongation and spindle organization are positively correlated with the mutant phenotype (Fig. S6B & E). The heat map comparison for genes involved in spindle organization and elongation are shown (Fig.S6). Several of the genes implicated in spindle organization have also been annotated under those for spindle elongation and hence appear under both categories. However, as shown in Fig.S3 relative enrichment of earlier stages compared to later stages limits the accurate prediction of altered pathways in *cpes* mutants.

Fig.S6. GSEA pathway analysis indicates enrichment of spindle elongation and spindle elongation processes. (A) GSEA pathway analysis of RNA seq data from w^{1118}, cpes mutant and bam-Gal4>UAS-CPES rescue testis. Three independent biological replicates were taken for each sample and RNA was extracted from 200 pairs of testes for each biological replicate. (B)

Enrichment plot showing genes involved in spindle elongation are positively correlated in cpes mutants compared to w^{1118} and bam-Gal4>UAS-CPES rescue. (C-D) Heatmap shows spindle elongation genes are enriched in cpes mutants. (E) Enrichment plot showing genes involved in spindle organization are positively correlated in cpes mutants compared to w^{1118} and rescue. (F-G) heat maps show the top 100 enriched genes in cpes mutants (H) heatmap shows the top 50 genes enriched in wild type and rescue.

3. In another related point, the claim that the RNAseq data are consistent with "a role [for sphingolipids] in regulating PIP2/PIP3 mediated signaling in vivo" (lines 577-588) is a bit of a stretch. There is an enrichment of spermatocytes in the cpes mutants and there is well documented EGFR (not mammalian ERBB) signaling between spermatocytes and cyst cells at this stage. The apparent upregulation of EGFR signaling likely has to do with the relative abundance of earlier vs. later stages of developing male germline cysts rather than a specific effect of sphingolipids. On the other hand, the pupal lethality of cpes mutants overexpressing PLCdelta-PH-EGFP, as reported in the methods (lines 607-610), suggests that PIP2 might be limiting in the cpes mutant, an observation that could be worth mentioning in the text.

We apologize for the abrupt introduction of the ERBB RNA seq data in this thread of discussion. The discussion is focused to a large extent about a potential role for sphingadienes in regulating PIP2/PIP3 signaling. Inadvertently, we highlighted the ERBB RNA seq data of the mutant in this discussion which is not consistent with how we sought to suggest a link between PIP2/PIP3 signaling and sphingolipids. We have removed the reference to ERBB in this context.

We agree with the reviewer that pupal lethality observed upon global over expression of PLCdelta PH-EGFP could indicate that PIP2 could be limiting in the *cpes* mutants. As suggested by the reviewer, the sentence has been edited to include this possibility.

Revised text: (lines 842-851)

Sphingadienes with $\Delta^{4,6}$ conjugated double bonds were previously identified in *Drosophila*, *Manduca sexta*, and *Bombyx mori* [66-68], although their enrichment in the testis was not known until now. However, normal sphingolipid synthesis and their degradation are shown to be required for spermatogenesis [69, 70]. The sphingadiene sphingolipids were shown to have inhibitory effects on AKT-dependent signaling [71] and downregulate *Wnt* signaling via a PP2A/Akt/GSK3β pathway [72]. Thus, accumulation of sphingadiene with saturated fatty acid containing sphingolipids in the testis could have a role in regulation of PIP2/PIP3 mediated signaling *in vivo*. Apart from CPE_SPD_SFA the *Drosophila* testis is also enriched in CPE_SPH_MUFA suggesting they could mediate distinct functions. Lipids with unsaturated fatty acids mediate a number of biological functions [73].

4. The images showing CPE colocalizing with Rab7 and Rab11 at the midzone of dividing spermatocytes, while tantalizing, fail to demonstrate that CPE is delivered to the furrow via endosomal trafficking. In particular, fusion of these endosomes with the furrow is not demonstrated. Therefore, it is unclear whether the endosomes actually deliver CPE to be incorporated into furrow membranes. Moreover, although Rab11 has been shown to be required for cytokinesis in Drosophila spermatocytes (Giansanti et al., 2007; Polevoy et al., 2009) and mammalian cells, a role for Rab7 in cytokinesis has not been demonstrated in any system. Thus,

to conclude that the presence of CPE-positive Rab7 and Rab11 at ingressing cleavage furrows "indicates their importance in delivery of lipids to the ingressing membranes of the furrow" is an overstatement. To be able to conclude this, CPE distribution would need to be examined in germ cells mutant for Rab11 or Rab7, or where these Rabs have been knocked down or dominant-negative versions have been expressed. In the absence of data showing a requirement for Rab11 and/or Rab7 in CPE delivery to the furrow, the title of the manuscript will need to be changed to something that better reflects the actual results, for example, "Ceramide phosphoethanolamine (CPE) with unique acyl chain anchors is essential for male meiotic cytokinesis."

Reviewers 1, 3 and 4 have suggestions and concerns regarding the delivery of CPE via the endosomes. In response to the comments and suggestions of the reviewers, we have performed experiments that have provided more insight into the role of endosomes in delivery of CPE to the ingressing furrow. Briefly,

1. We have performed CLEM/ FIB-SEM (correlative light and electron microscopy (focused ion-beam-scanning electron microscopy) of Rab7 marked endosomes. In one set of experiment, we discovered that these Rab7 and CPE co-labeled structures are multivesicular endosomes and migrate to the cleavage furrow during cytokinesis. Using CLEM/FIB-SEM we focused on those vesicles that are in close proximity to the ingressing membranes and discovered that the multivesicular endosomes are tethered to the ingressing membranes of the cytokinetic furrow. Further analysis using CLEM-transmission electron microscopy showed that the intraluminal vesicles within the MVBs were being deposited at the cleavage furrow in close proximity to the ingressing

membranes, perhaps providing a source of membrane components for the newly formed and expanding plasma membrane of meiotically dividing cells.

2. As suggested by reviewer 1, we evaluated meiotic cytokinesis in Rab7 mutants and dominant negative Rab11 mutants. We find between 15-25% of the spermatocytes fail to undergo meiotic cytokinesis in Rab7 mutant indicating a small but significant role for Rab7 (Figure S7). Our observations show that CPE positive endosomes continue to form and cycle to the furrow in the Rab7 mutant indicating existence of redundant mechanisms (Movie S13). Rab11 mutant are embryonic lethal and therefore we used conditional expression of dominant negative Rab11 mutants and discovered significant cytokinesis defect in them (Figure S7).

3. GENERAL COMMENTS

1. "Meiotic cytokinesis" is incorrectly referred to as "meiosis cytokinesis" in many places in the text. This should be corrected throughout.

We thank the reviewer for the corrections and suggestions.

We have now corrected "meiosis cytokinesis" to "meiotic cytokinesis" throughout the manuscript.

2. Adding a description and figure of the entire CPE synthesis pathway to the Introduction would help readers who are unfamiliar with this class of lipids and how they are produced.

We have now included a figure for the CPE synthesis pathway in Figure 1 as Panel A.

3. It would help to highlight particular lipid species of interest, especially those that are enriched in testes, in the tables in Data S1 and S2 to aid the reader in finding the relevant data.

We have now highlighted the CPEs of interest in blue in tables in Data S1 and S2.

4. Several relevant references are missing. In addition to the Rab11 references listed above, papers describing des-1 mutants defective in ceramide synthesis that show spermatocyte degeneration and meiotic defects (Endo et al., 1996; Basu and Li, 1998) could be discussed in light of the meiotic defects observed in cpes mutants.

We thank the reviewer for the suggestion and have included the references. The discussion has been edited to include des-1 mutants.

The following passage has been added to the discussion (lines 854-866)

des-1 codes for dihdryoceramide desaturase enzyme in the *de novo* biosynthetic pathway in *Drosophila*. *des-1* mutants (also called *infertile crescent*, *ifc*) were described before the gene product was identified as a homolog of dihydroceramide desaturase. The primary spermatocytes of the *des-1* mutants undergo degeneration without initiating chromosome condensation at the beginning of meiosis [76]. A P-element insertion allele showed that DES-1 colocalized with mitochondria and was intimately associated with the central spindle of dividing meiotic spermatocytes. Its deficiency leads to failure of central spindle assembly in these dividing cells leading to several phenotypes including cytokinetic defects. It was proposed that DES-1 could be part of an anchoring mechanism that linked membrane bound cellular compartments to components of the cytoskeleton [69]. Considering that DES-1 is a dihydroceramide desaturase

homolog, it would be worth revisiting the mutant phenotype to evaluate if it too contributes to membrane assembly at the cytokinetic furrow due to lack of CPEs.

5. The writing needs to be carefully edited for grammar, punctuation, spelling and pithiness.

We have edited the manuscript and hopefully has fewer grammatical errors

6. Abbreviations need to be defined the first time they appear (MRM, CPMT, ReTS).

We have defined the abbreviations noted by the reviewer.

SPECIFIC COMMENTS

Text:

line 1: delete "Endosome mediated delivery of" (see Major Comment 4)

We have edited the text substantially and have deleted 'Endosome mediated deliver of…" The new title reads as "**Endosomes deliver ceramide phosphoethanolamine (CPE) with unique acyl chain anchors to the cleavage furrow during male meiotic cytokinesis"**. The manuscript and the title have been edited to accommodate findings from the new experiments.

lines 2-3: replace "meiosis cytokinesis" with "meiotic cytokinesis" (here and throughout)

We have replaced 'meiosis cytokinesis' with 'meiotic cytokinesis" here and throughout in other places (line 2)

line 21: replace comma with semicolon after "midbodies"

Reviewer 2 has suggested the use of the term intercellular bridge instead of midbody. A semicolon replaces the comma after the term "intercellular bridges" (line 26)

line 25: substitute "mislocalization" for "localization"

We have edited the sentence to read as mislocalization (line 31)

line 25: replace "phosphatidyl inositols" (also "phosphatidylinositols", e.g., line 45) with phosphatidylinositol phosphates (PIPs); can use PIPs after the first time this is defined

We have made the suggested changes (line 31-32)

line 35: delete comma after "species"

Done (line 46)

line 46: "groups" should be pluralized

We have changed the word to “groups” (line 54)

lines 57-58: awkward as written; suggest changing to "Cell division, wherein one cell divides into two daughter cells, is fundamental to all living organisms."

We have made the suggested changes (lines 68)

line 58: delete "the" before "cytokinesis"

Thank you. We have deleted “the” before “cytokinesis” (line 69)

line 75: fix typo in "polyunsaturated"

We apologize. We have corrected it (line 84)

line 90: add comma after "Fig.1A"

Old Fig. 1A is the new Fig. 1B. A comma has been added after “Fig. 1B” (line 108)

line 93: delete "the" before "heads"

We have made the change (line 111)

line 103: add colon after "properties"

We have added the colon (line 130)

line 112: delete "s" in "comparisons" (here and elsewhere, e.g., line 181)

We corrected it here and other places too and we also corrected this error in other places (lines 139, 161, 226, 227, 230, 337, 478)

lines 138, 139, 141: replace "Hexosylceramides and its subspecies" with "Hexosylceramide subspecies"

Done. Thank you (lines 161, 165, 166, 168, 228, 471)

line 152: delete "s" in "sperms"; also, seminal vesicles are not shown

We have deleted ‘s’ in “sperms” and changed the text to (in lines 182-183)

“Mature sperm in seminal vesicles (data not shown).”

line 163: "nebenkern" is misspelled

“nebenkern” has been corrected (line 189)

line 164: replace "is" with "are"

We have replaced the “is’ with “are” (line 190)

line 178: capitalize "Whole"

Done (line 208)

line 191: replace "nucleus" with "nuclei" and pluralize "tails" and "axonemes"

We have replaced “nucleus” with “nuclei” and pluralized “tails” and “axonemes” (line 234)

line 200: insert "the" before "individualization"

We have included “the” before “individualization” (line 242)

line 209: insert "cleaved" before "Drosophila Caspase 1"

We have now included “cleaved” before “Drosophila Caspase 1” (line 251)

line 218: replace "individuating" with "individualizing"

We have now replaced "individuating" with "individualizing" (line 263)

line 221: would be clearer to use "Bam-Gal4" and "UAS-CPES" (with hyphens) here and elsewhere

Done. Thank you (line 266, 285, 294, 296, 309, 320, 332, 338, 349, 356 etc)

line 222: for flow, it would help to move the sentence starting with "The mutant and rescue samples (lines 224-226) immediately after "(Data S1)."

We have moved this sentence to after Data S1 (lines 267-268)

line 223: remove "s" from "germ"

Done. Thank you (line 270)

line 224: pluralize "levels"

We have pluralized "levels" (line 271)

lines 234-235: use subscripts for the amino acid motif, similar to what is shown in line 239 (i.e., DX2DGX2, etc., where the numbers are subscripts)

Thank you. We have edited to now using the motif with subscripts (line 279-280)

lines 244-247: the legend does not correspond to what is in the figure: "(A-C)" should be replaced with "(A, C, E)", "(B)" should be replaced with "(C)" and "(C)" should be replaced with "(E)"; panels B, D, and F are not described

We have now edited the legends to correctly describe the supplementary figure S2. We apologize and thank the reviewer for highlighting this error.

Revised manuscript (lines 288-96)

Fig.S2. CPES enzymatic activity is required for spermatogenesis and accumulation of ceramide is not responsible for cpes mutant phenotypes. *(A, C and E) Live testis squash preparation followed by phase contrast microscopy of round spermatids, (B, D and F) are immunofluorescence*

images of early elongating spermatids from samples corresponding to A, C and E respectively. Immunostaining was performed with beta tubulin primary antibody and Alexa Fluor 568 conjugated secondary antibody (Red). Nuclei are stained with DAPI (Blue) (A and B), the UAS-CPES active site mutant (GX_3DX_3D to GX_3AX_3A) was expressed using bam-Gal4 in cpes mutant background. (C and D), cpes; dcert[1] double mutants. (E and F) The UAS CDase (neutral ceramidase) was expressed using bam-Gal4 driver in cpes mutant background.

line 255: replace "testis was" with "testes were"

We have replaced “testis was” to “testes were” (line 303)

line 258: for clarity "in the ER" should be added after "accumulation"

We apologize for lack of clarity here. We have edited the sentence to make the point clear that accumulation of ceramide in the Golgi complex in a cpes mutant was probably not responsible for the phenotype.

Revised manuscript (lines 304-307)

However, as shown in Fig.S2C and D, *cpes*; *dcert[1]* double mutants did not rescue meiotic cytokinesis and spermatid polarity indicating ceramide accumulation at the Golgi Complex (due to lack of CPES activity in the Golgi) may not be responsible for *cpes* mutant phenotypes.

line 259: remove "s" from "reduce"

Thank you. We removed “s” from “reduce” (line 308)

line 260: delete "have" and remove "s" from "germ"

We have removed “s” from “germ” (line 309)

line 261: "the progeny testis was" doesn't make sense; replace with "testes from the resulting progeny were"

Thank you. We have now edited the sentence to read as “testes from the resulting progeny were”.

Revised text (lines 308-310)

We overexpressed ceramidase (UAS CDase) in germ cells using *bam*-Gal4 in *cpes* mutant background and testes from the resulting progeny were analyzed for the rescue of mutant phenotypes.

line 262: delete "again"

We have deleted “again” (line 311)

lines 264-265: could delete sentence beginning with "Also", as this is addressed later on

We have deleted the sentence beginning with “Also” (line 313)

line 271: replace "and" with "or"

We have replaced “and” with “or” (line 346)

line 280: insert "and" after "cytokinesis" and a comma after "polarity"

We have added “and” after “cytokinesis” and a comma after “polarity” (311)

line 283: delete the comma and "the" after "Although"

We have deleted the comma and “the” after “Although” (line 384)

line 287: delete "The"

We have deleted “The” (line 388)

line 289: insert "cell" after "4-16"

We have added “cell” after “4-16” (394)

lines 293-297: this section is redundant with the earlier part of the figure legend

We have deleted the earlier section that was redundant.

line 304: delete "the" after "performed", pluralize "tests" and delete "as described in the methods section"

We have deleted “the” after “performed”. We have pluralized “tests” and deleted “as described in the methods section”. (line 420)

line 310: replace "expression" with "localization"; also, explain here or in the legend how CPES-V5 was expressed

We have replaced “expression” with “localization” (line 468). We have now included the following text in the legend about CPES-V5 expression.

Revised manuscript (lines 425-432)

To determine *in vivo* CPES localization more precisely at the protein level, we have generated a construct with a 6437 bp extended CPES genomic fragment that was modified by recombineering to carry V5-tag at the C-terminus [15]. Transgenic flies expressing genomic CPES tagged with V5 at the C-terminus fully rescued lethality, photosensitive epilepsy and all other phenotypes including spermatogenesis defects. Immunostaining followed by confocal imaging showed that CPES_V5 was highly expressed in the cells that are present just below the tip of the testis (Fig.3P and S). This area of the testis is known to be enriched in late spermatogonia and early spermatocytes [17].

lines 370-371: in Fig. 4A and 4E, the driver is not actin-Gal4, so it is inaccurate to say "yet at this level of expression we did not see rescue"

This data is now part of Figure S5. We have edited that sentence to include reference to tubulin.

Revised manuscript (lines 456-459)

Fig.S5. Insect CPE synthases but not sphingomyelin synthases rescue spermatogenesis defects. *(A-C) Phase contrast microscopy of round spermatids where UAS hSMS1 (A), UAS Aedes CPES (B), and UAS Bombyx CPES (C) were ubiquitously expressed in cpes mutant background using tubulin Gal4. The scale bar is equivalent to 10 μm.*

line 390: "several" is an understatement; a lot of genes were enriched

We have changed the sentence to read as (lines 515-517)

GSEA showed many genes involved in spindle elongation and spindle organization are positively correlated with the mutant phenotype (Fig. S6B & E).

lines 392-393: need to insert description of Data S3 and S4 here

Data S3 and S4 has been reorganized and included in Figure S6.

Revised text: (Lines 515-521)

GSEA showed many genes involved in spindle elongation and spindle organization are positively correlated with the mutant phenotype (Fig. S6B & E). The heat map comparison for genes involved in spindle organization and elongation are shown (Fig.S6). Several of the genes implicated in spindle organization have also been annotated under those for spindle elongation and hence appear under both categories. However, as shown in Fig.S3 relative enrichment of earlier stages compared to later stages limits the accurate prediction of altered pathways in *cpes* mutants.

line 411: replace “leading to the” with “led to”

We have replaced “leading to the” with “and led to” (line 549)

line 422: for clarity, replace the sentence starting with “horizontal” with “In horizontal view (left),…” and start a new sentence after “shown,” starting with “In cross-sectional view…”

We thank the reviewer for the suggestion. We have incorporated the suggested edits. (lines 560-562)

line 427: delete “expressing”

We have deleted the word “expressing” (line 564)

line 439: delete “s” in “PIP”

We have deleted “s” in “PIP” (line 574)

lines 441 and 443: use of “Tag:PH” for these transgenes is confusing to a non-expert, as this is Flybase-specific nomenclature; use instead “EGFP fused to the PH domain of human PLCdelta1” and “EGFP fused to the PH domain of Steppke”

Thank you. We have now expanded the sentence to incorporate the reviewer’s suggestion.

Edited sentence (lines 579-583)

To investigate PIP distribution in *cpes* mutant membranes we used PIP2 reporter UAS PLCδ-PH-EGFP (UAS regulatory sequence drives expression of EGFP fused to the PH domain of human PLCdelta 1) and PIP3 reporter tGPH (an alphaTub84B promoter drives the expression of EGFP fused to the PH domain of Steppke) [43, 44].

lines 497-498: reword to delete "indicates their importance in delivery of lipid to the ingressing membranes of the furrow", as this is an inference not supported by the data (see Major Comment 4 above)

Following the earlier suggestion of the reviewer we have now performed genetic and CLEM/FIB-SEM and CLEM/TEM experiments to highlight the importance of CPE trafficked by the endocytic pathway. A modified sentence has now been included after lines 637-640 “Overall, the endocytosis of CPE and targeting to the ingressing cleavage furrow *via* Rab7 and Rab11 marked endosomes indicates their importance in delivery of lipids to the ingressing membranes of the furrow and coordination of this event with proper spindle behavior and meiotic cytokinesis.”

line 503: fix typo in "spermatocytes"

We have fixed the type in “spermatocytes” (line 650)

line 507: "Arrowheads" is one word"

We have merged “Arrowheads” as one word (line 681)

line 511: replace "were" with "are:

We have replaced “were” with “are” (line 690)

lines 512 and 513: replace "was" with "is"

We have replaced “was” with “is” (line 692)

line 520: delete "s" from "remain"

We have deleted “s” from “remain” (792)

lines 525 and 529: clarify what is meant by "mitotic cells"; in what organism or cell type?

We apologize for the ambiguity. This study was performed in HeLa cells. We have accordingly edited the sentence.

Revised text: (lines 797-799)

In mitotic HeLa cells, PIP2 accumulation at the inner leaflet and subsequent recruitment of RhoGTPase (RhoA) was shown to be coordinated with sphingomyelin rich domains on the outerleaflet [10].

line 538: replace "complement for" with "substitute for"

We have replaced “complement for” with “substitute for” (line 810)

line 553: add period after cytokinesis and start next sentence with "In particular,"

We have added a period after cytokinesis. We have started the new sentence with “In particular” (line 825)

line 555: replace "do" with "does"

We have replaced “do” with “does” (line 827)

line 556: add period after "furrows" and start next sentence with "Instead,"

We have added a period after “furrows”. We started the next sentence with “Instead,” (line 828)

line 561: rewrite to clarify that Steppke itself was not examined, only its PH domain

The text has been edited to indicate that “Steppke” itself was not examined but rather only its PH domain. It has been edited to as follows:

Revised text (line 832-834)

Intriguingly, we show that the PIP3 binding PH domain of Steppke (tGPH) localizes to cleavage furrows indicating a potential role for PIP3 during male meiotic cytokinesis.

line 569: clarify what is meant by "functional alterations" (of what?)

We apologize for the ambiguity. The text has been edited and reads as follows:

Revised text (lines 839-842)

Although broad accumulation of PIP2 to the plasma membranes and PIP3 accumulation at the cleavage furrows was not altered in *cpes* mutants, their specific localization to membrane microdomains and their inability to signal robustly cannot be ruled out.

line 584: insert "The" before "human genome"

We have inserted "The" before "human genome" (line 881)

line 687: replace "Testis" with "Testes" and "tore" with "torn"

We have replaced 'Testis" with "Testes" and "tore" with "torn" (996)

line 689: replace "opened" with "open", add a period, and start a new sentence with "They"

We have replaced "opened" with "open". We have added a period. We started a new sentence with "They" (line 998)

Figures:

Fig.1E: fix typo; "WTTT" should be "WTTS"

We have changed "WTTT" to "WTTS"

Fig. 2M-O: upper labels ("WT MT RES_CPE", etc.) need to be defined in the legend or omitted; also, CPMT and ReTS need to be defined in the legend or in the figure

We have corrected these errors. These changes are as follows:

Revised text in the legends

Upper labels (WT MT RES_CPE) were omitted in the figure and the legends was edited to include CPMT and ReTS.

"(M-O) Sphingolipid analysis in lipid extracts of dissected testis, wild type testis (WTTS);cpes mutant testis (CPMT)and Rescue testis (ReTS).

Fig. 3O-R: it would help to show insets demonstrating colocalization of V5-CPES with GM120 at the Golgi

We have included an inset showing colocalization of V5-CPEs and GM130.

Fig. 4J: "T. Urticae" should be written "T. urticae"; it would be worth commenting in the text whether the putative T. urticae CPES might have some other activity since it does not rescue

Since the tick CPES did not rescue and we have not investigated the gene product in depth we have removed it from the revised manuscript. This was also in part as a response to reviewer 4 comments. Also, we have now moved the data from rescue experiments using mosquito, silkworm CPES and human SMS1 to supplemental section.

Fig. 5A: it would help to indicate in the panel or legend that the GSEA analysis shows gene sets enriched in cpes mutants relative to controls

Based on previously discussed comments from the reviewer the RNAseq data has been reanalyzed and submitted as Fig. S3 and Fig. S6.

Fig. 5B-C: it would help to label several genes as examples in each plot, especially since it is hard to correlate what is plotted here with the data shown in the supplementary datasets

In the revised figures, we have shown heat maps that have been enlarged, categorized, and laid out in a more discernible fashion (Fig. S3 and Fig. S6).

Fig. S1: it would help to include a diagram of hexosyl ceramides similar to what is shown for CPE in Fig. 1D

We have drawn a diagram of hexosylceramide in Fig. S1

Fig. S1A: the order of samples in the key should correspond to the order shown (WTHM, WTHF, WTOY, WTTS), as was done for the other panels (Fig. SB-D)

We have corrected the order of the samples in key to correspond to the order shown in the figures.

Rev. 2:

Kunduri and co-workers present a study of the role of ceramide phophoethanolamine during the incomplete cytokinesis that occurs during Drosophila spermatogenesis. The overwhelming focus of the cytokinesis field is on the role of proteins, but a small number of studies demonstrate that lipids have vital roles. However, our understanding of how lipids participate in cytokinesis is scant. Using fly genetics combined with elegant microscopy, Kunduri and co-workers establish new insight into the role of specific lipids in cytokinesis and how these lipids are delivered to the cytokinetic furrow. This will be an appreciated study in the field.

I only have some minor comments.

1. line 58 "the cytokinesis" should be changed to "cytokinesis"

Thank you. We have changed “the cytokinesis” to “cytokinesis” (line 68)

2. line 62 and 69. I hope this doesn't seem too picky and it is not necessarily the fault of the authors, rather the field, but a consistency in nomenclature should be applied. On line 62 the authors use the term "midbody" but on line 68-69 use "intercellular bridge" I think they refer to the same structure. Over the years midbody has been used in different studies to describe different structures, sometime the connecting bridge, sometimes the bulge in the middle of the bridge (also referred to the stem body or the Flemming body). It would be good for the authors to pick one and stick to it and define it. My preference is to use intercellular bridge to describe the whole connecting structure between the two cells, but really it is at the discretion of the authors. In light of that it may also be useful and helpful to the reader to have a diagram / cartoon / model

to summarize the findings at the beginning and the end (the more important spot) to help distill the findings and put forth a model clear to the reader, especially a non fly person.

We have followed the reviewer's suggestion and replaced midbody with intercellular bridge throughout the manuscript. We have also included a model to summarize the finding at the end of the manuscript (Fig. 8E)

3. on line 150 it would help the general reader to have a concise summary of what the cpes mutant is i.e. what enzyme is affected etc. That will lead the reader in nicely to the experiments the authors perform.

Thank you. We have now included a short description about the *cpes* mutant. This was also suggested by reviewer 1 and we thank both reviewers for seeking clarity on this issue since it will facilitate an easier reading of the manuscript.

Revised manuscript: (lines 89-93)

Previously, we have shown that *cpes* null mutants show significant late pupal lethality during development and only about 25% of mutants survive to adulthood. About 60% of *cpes* mutant adults have dorsal closure defects and all the mutant males are sterile. Aged *cpes* mutant adults display light inducible seizures and paralysis due to defective cortex glia [15].

4. On line 268 "we have performed" should be "we performed"

We have corrected “we have performed” to “we performed” (line 341). We have also corrected similar statements throughout the manuscript. Thank you.

5. Either when first used or in the discussion it would help the non fly person to have a more

detailed discussion of the difference between the nos and bam promotors. Could anything more be learnt form their differences? Could this tie in further with the spatio-temporal studies and conclusions from it?

We have now included a short discussion about *nos* and *bam* promoters and the possible inferences drawn from our studies using drivers driven by the two promoters. Accordingly, the discussion incorporates the following changes.

Revised manuscript (lines 349-362)

Nanos (*nos*) gene encodes for an RNA-binding protein involved in formation of translational repressor complex. It is required for germ plasm organization, germline development, germline stem cell renewal and neuronal morphogenesis [35]. To specifically express a transgene of interest in male and female germline stem cells *nanos*-Gal4-VP16 was routinely used. This construct consists of 700bp *nos* promoter, Gal4-VP16 ORF, *nos* 3' UTR and 500bp of 3' genomic *nos* transcription unit. In male germ line *nos*-Gal4 primarily expresses in GSC and early spermatogonial stages [36]. Bag of marbles (*bam*) gene encodes for a fusome (a germ cell specific organelle) associated protein. The Bam protein is required for activation of the switch from spermatogonia to spermatocytes. The construct *bam*-Gal4-VP16 has been successfully used to drive the expression of transgenes in late spermatogonia and early spermatocytes in *Drosophila*. This construct consists of 900bp bam promoter, 500bp *bam* 5'UTR, Gal4:VP16 ORF and HSP70 3'UTR. Although transgenes driven with *bam*-Gal4-VP16 express in late spermatogonia through early spermatocytes, the peak expression was shown to occur during spermatocytes [37].

6. Can anything more be learnt or discussed from a more detailed comparison of the spider mite enzyme (Fig 4) with the enzyme from other species? Sequence differences, localization , enzyme activity etc that may help explain the differences observed and the failure of the spider mite enzyme to rescue the cpes mutant?

We have not explored the enzymatic activity of the spider mite CPES homolog in detail and this has created ambiguities as noted by the reviewer 2 and also reviewers 1 and 3. Therefore, we have deleted the data corresponding to spider mite CPES expression and have moved rest of the data from mosquito, silk worm CPES homologs and the human SMS to the supplemental section as suggested by reviewer 4. Thus, Figure 4 is now Figure S5. Accordingly, the following changes have been incorporated in the manuscript.

Revised manuscript (lines 436-502)

Insect CPE Synthases but not Sphingomyelin Synthase rescue male sterility defects

Previously we have shown that CPE has an important structural role in cortex glial plasma membranes [15]. The cortex glial plasma membranes are severely defective in *cpes* mutants leading to loss of cortex glia neuronal cell body interactions during development and increased susceptibility to light inducible epilepsy in adults. Further, we have shown that overexpression of human sphingomyelin synthase 1 (hSMS1) that produces SM instead of CPE was sufficient to rescue cortex glia plasma membrane defects via establishment of detergent resistant membranes. We wondered whether overexpression of hSMS1 could also rescue the spermatogenesis phenotypes of *cpes* mutant. To this end, UAS-hSMS1 was ubiquitously expressed using tubulin-Gal4 in *cpes*, the adult male testes were dissected, and live testis squash preparations were observed using phase contrast microscopy. However, as shown in Fig.S5A, hSMS1 overexpression did not rescue male meiotic cytokinesis. Further, immunostaining analysis showed

that spermatid polarity was also not rescued (Fig.S5E) suggesting an important role for the CPE head group in spermatogenesis. We next investigated whether expression of other insect derived CPES could rescue *cpes* mutant phenotypes. To test this, we ubiquitously expressed *Aedes aegypti* (Yellow fever mosquito, amino acid sequence shows 60% identity and 77% similarity), and *Bombyx mori* (domestic silk moth, amino acid sequence shows 43% identity and 58% similarity) derived UAS-CPES homologs in *cpes* mutant background using tubulin-Gal4. As shown in Fig.S5 mosquito and silk moth CPES were able to completely rescue defects in meiotic cytokinesis (Fig.S5B-D) and spermatid polarity (Fig.S5F and G respectively).

Fig.S5. Insect CPE synthases but not sphingomyelin synthases rescue spermatogenesis defects. *(A-C) Phase contrast microscopy of round spermatids where UAS hSMS1 (A), UAS Aedes CPES (B), and UAS Bombyx CPES (C) were ubiquitously expressed in cpes mutant background using tubulin Gal4. The scale bar is equivalent to 10 µm. (D) Measurement of cytokinetic defects in UAS-hSMS1, UAS-Aedes aegypti and UAS-Bombyx mori CPES rescue testes. 4 independent experiments for UAS-hSMS1 (round spermatid count, n=500), 2 independent experiments for UAS-Aedes aegypti (round spermatid count, n=1100) and 4 independent experiments for UAS-Bombyx CPES (round spermatid count, n=1900). (E-G) Confocal images of elongating spermatids immunostained with beta tubulin primary antibody and Alexa Fluor 568 secondary antibody (Red) and DAPI for DNA (Blue). The UAS hSMS1 (E), UAS Aedes CPES (F) and UAS Bombyx CPES (G). (H-K) Sphingolipid analysis of lipids extracted from testis of wild type, cpes mutant, and various rescues expressed in germ cells using vasa-Gal4. In contrast to Figs.1 and 2, here we used whole male organ (testis, accessory glands, collecting ducts and ejaculatory bulb) for lipid extraction. (H) The amounts of CPE and its subspecies in wild type, cpes mutant and various rescues are shown. (I) Ceramide and its subspecies are shown in picomoles per 100µg of carbon.*

*(J) Hexosylceramide subspecies amounts are shown in bar diagram. (K) Amount of sphingomyelin (SM) and its subspecies are shown as picomoles per 100µg of carbon. Fluorescent blue green part of the bar represents SM species with net two double bonds in them. (L) Thin layer chromatography (TLC) of lipids extracted from 50 pairs of testes from each genotype. The position of SM on TLC is indicated by an arow. Dihydrosphingosine (DHSPH), Sphingosine (SPH), Sphingadiene (SPD) Saturated fatty acid (SFA), Monounsaturated fatty acid (MUFA) and polyunsaturated fatty acid (PUFA). Statistical significance was calculated using mean, standard deviation (SD) and N in Prism 8. The 2way ANOVA multiple comparison was used to calculate P values where ****P ≤ 0.0001;***P ≤ 0.001; **P ≤ 0.01; *P ≤ 0.05 and ns P >0.05. Three independent biological replicates were taken for each sample and lipids were extracted from 250 male organs for each biological replicate.*

To investigate the nature of sphingolipid species synthesized by hSMS1, *Aedes aegypti*, *Bombyx mori* derived CPES, we specifically expressed these transgenes in germ cells using *vasa*-Gal4, their male reproductive system was dissected, lipids were extracted and subjected to sphingolipid analysis using SFC/MS/MS as described in the methods section (Data S2). As shown in Fig.S5H germ cell specific expression of *Drosophila* CPES, *Bombyx mori* CPES completely restored CPE to the wild type amounts. *Aedes aegypti* CPES also synthesized significant amount of CPE but to a lower amount than *Drosophila* CPES. However, all three distinct CPE species are synthesized by *Aedes aegypti* CPES and *Bombyx* CPES (Fig.S5H). As anticipated, hSMS1 did not synthesize CPE (Fig.S5G but produced significant amount of SM (Fig.S5K). However, the amount of SM is relatively low compared to CPE, perhaps due to limited expression/stability of hSMS1 in germ cells (vasa-Gal4). Indeed, ubiquitous expression of hSMS1 using actin-Gal4 driver significantly increases the SM amounts (Fig.S5L). However, expression of hSMS1 with ubiquitous drivers like

actin-Gal4 and tubulin-Gal4 did not rescue the spermatogenesis (Fig.S5A and E; data not shown for actin-Gal4) suggesting the importance of the CPE head group. We next analyzed the ceramide content in these samples and found that ceramide levels were significantly high in *cpes* mutants and hSMS1 rescue compared to *Drosophila* CPES rescue. However, as discussed in Fig.S2 higher ceramide content might not contribute to the cytokinetic defects in *cpes* mutants. Similarly, higher amounts of HexCer in *Aedes aegypti* CPES rescue and *Bombyx mori* CPES rescue also did not correlate with the rescue of spermatogenesis phenotypes (Fig.S5J, B, D, & F). Taken together these results suggest that the *Drosophila* spermatogenesis is strictly dependent on CPE and the head group of CPE play an important role.

7. On line 410 is the description of the mutant correct? My interpretation of the movies and images in Fig 5D is that in the mutant there is barely any furrow ingression at all. The microtubules bundle and compact, whereas the membrane appears to barely ingress and therefore there is no regression.

We agree with reviewer that there is barely any furrow ingression. We apologize that our construction of the sentences did not convey this sentiment. We have rewritten the sentences to convey that there is barely any furrow ingression in the *cpes* mutant spermatocytes. Accordingly, the following changes have been incorporated.

Revised manuscript: (line 547-556)

Interestingly, in *cpes* mutants, the initial assembly of central spindle and actomyosin ring occurred normally (Movie S3) however, central spindle and actomyosin ring constriction became uneven since there is barely any furrow ingression and led to substantial disengagement between

the contractile ring and the central spindle during meiosis (Fig.4A). As a result, the central spindle and actomyosin ring disassembled prematurely (Movie S3). Quantification of this behavior suggested that more than 80% of the spermatocytes showed detachment of the contractile ring and central spindle (Fig.4B). Other minor variations included the contractile ring detaching from the plasma membrane, contractile ring sliding and dumping to one end and the contractile ring and central spindle being destabilized simultaneously at one end (Fig.4B). Expression of UAS CPE in germ cells was sufficient to rescue this phenotype (Movie S4, Fig.4A).

Rev. 3:

In their manuscript, Kunduri and colleagues analyzed the function of cpes in Drosophila spermatogenesis. cpes encodes for ceramide phosphoethanolamine synthase, which is required for lipid membrane biogenesis. The authors previously showed that males KO for cpes were partially viable and sterile (Kunduri, PNAS, 2018). Here, they showed that the sterility is caused by defects during cell divisions of germ cells. They correlate this defect with specific lipid composition of male germ cell which they uncovered using SFC/MS/MS (mass spec methods).

The descriptive parts of the manuscript are really nice. The authors demonstrate specific lipid compositions in testis vs head vs ovaries. Descriptions of phenotypes in testis are very convincing and movies are impressive.

My criticisms regard mostly the functional parts. I understand that the authors want to make the link between specific lipid composition in male germline cells and specific phenotypes in cytokinesis in cpes mutants. However, it is not clear whether cytokinesis defects are also present in other organs with different lipid compositions. Only 25% of cpes mutant flies are viable (Kunduri, PNAS, 2018): are there cytokinesis defects in ovaries? in heads? or any other organs?

We thank the reviewer for suggesting additional control experiments. We have now performed the following experiments. We have examined mitotic cytokinesis in larval neuroblast (Movie S1) and do not find cytokinesis defects in these cells. Females are largely fertile indicating no defects in oogenesis. Further, even during spermatogenesis, we do not observe any defect during

the formation of 16 cell staged spermatocytes indicating that mitotic cytokinesis even in testis of *cpes* mutants is not compromised.

We have now included all this information in the text.

Revised manuscript: (lines 195 – 206)

As shown in Fig.2D, wild type round spermatids contained an equal sized phase light nucleus and a phase dark nebenkern at a ratio of 1:1 per cell within the cyst of 64 cells. In contrast, *cpes* mutant round spermatids showed four regularly sized nuclei (tetranucleate) and one abnormally large nebenkern, indicating a failure in cytokinesis in both meiosis I and II divisions (Fig.2E). Quantification of this defect suggest 80% of the round spermatids show tetranucleate and about 20% show two nuclei (Fig.2M). We did not observe single nucleated round spermatids *in* cpes mutant testes (Fig.2M). This defect is unique to male meiotic cytokinesis since larval neuroblasts undergoing asymmetrical mitotic division do not show defects in cytokinesis (Movie S1). Females are largely fertile indicating normal oogenesis (data not shown). Also, even during spermatogenesis we do not observe any defect in the formation of 16 cell staged spermatogonia/spermatocytes indicating that mitotic cytokinesis even in the testis of *cpes* mutants, is not compromised.

The link between RNAseq results in cpes mutant and spindle detachment from the actomyosin ring is also very unclear to me. I don't think that is has been shown that cytokinesis was regulated at the transcription levels and it could be very indirect.

We do agree with both reviewers 1 and 3 that our study has not been able to extract information about mechanistic insight into the pathology underlying *cpes* phenotype. As suggested by

reviewer 1 we have now rewritten RNAseq results and presented it as data supporting cytological and structural findings noted from other experiments described in the manuscript. In the revised version of the manuscript the RNA seq data has been moved to the supplemental section. As suggested by reviewer 1 we have reanalyzed the data and the information gleaned from the RNAseq experiments. The data was analyzed for the top 50 genes that were enriched in individual cell types as described by Witt. D et al (eLife 2019;8: e47138 DOI:10.7554/eLife.47138). The analysis indicated that gene expression largely complementing the phenotypes obtained from imaging experiments i.e., there was a general enrichment for genes expressed in the earlier stages of spermatogenesis and a downregulation of genes expressed in the post-meiotic stages of spermatogenesis (Fig. S3). The narrative has been appropriately edited to indicate that analysis of RNAseq data is supportive of the observed phenotypes broadly. We have avoided sentences that imply RNAseq analysis was performed to extract mechanistic insight.

Revised manuscript: lines 314-339

Transcriptomic and genetic dissection of spatiotemporal role of CPES in spermatogenesis

Spermatogenesis is a highly conserved process across various taxa that is facilitated by highly dynamic transcriptome. An earlier study leveraged single cell RNAseq and unsupervised clustering to identify all the major cell classes of the sperm lineage and validated them with previously studied marker genes [33]. To characterize cell type specific transcriptional signatures in *cpes* mutants *in vivo*, we performed total RNA-seq with dissected testis from w^{1118}, *cpes* mutant and *bam*-Gal4>UAS CPES rescue. Using Gene Set Enrichment analysis (GSEA) we compared our RNAseq results with the top 50 expressed genes from each cell type/cellular status that were

previously classified to confirm the presence of germline stem cells (GSC), spermatogonia, spermatocytes, and spermatids (germ cells) as well as cyst stem cells, terminal epithelial cells and hub cells (somatic cells) [33]. We found that gene sets corresponding to GSC, early spermatogonia and late spermatogonial cells are enriched in *cpes* mutants compared to wild type and rescue testis (Fig.S3). In contrast, gene sets corresponding to early and later spermatocytes, early, and mature spermatids are enriched in wild type and rescue testis compared to *cpes* mutant testis. These results suggested that CPES might play important roles during late spermatogonial, early and late spermatocyte stages for successful completion of spermatogenesis.

Fig.S3. Analysis of cell type specific transcriptomic signatures in cpes mutants: *(A) GSEA using top 50 cell type specific expressed gene sets and compared between cpes versus wild type (WT) and bam-Gal4>UAS CPES rescue (Rescue). Red bars indicate gene sets enriched in cpes mutants and blue bars indicate gene sets enriched in WT & Rescue. (B) enrichment plots for each of the cell type shows the distribution of individual genes in each set (vertical lines on red to blue parallel bar). Genes with positive enrichment score (ES) indicate that they are enriched in cpes mutants whereas negative enrichment score indicate that they are enriched in WT and Rescue. (C) Heatmap comparison of cell type specific enriched gene sets in w wild type (W1118), cpes and Rescue (bam-Gal4>UAS CPES rescue). Red indicates gene sets enriched in cpes mutants and blue indicate gene sets enriched in WT & Rescue.*

Finally, the localization of endosomes containing specific lipids to the cleavage furrow is very interesting, but it does not demonstrate that these lipids are required for proper cytokinesis. In addition, these PlyA2-positive endosomes are still present after cell divisions, and do not seem to

have fused with the cleavage furrow. It is thus not clear if anything has been "delivered" to the furrow.

Reviewers 1, 3 and 4 have suggestions and concerns regarding the delivery of CPE via the endosomes. In response to the comments and suggestions of the reviewers, we have performed experiments that have provided more insight into the role of endosomes in delivery of CPE to the ingressing furrow. Briefly,

1. We have performed CLEM/ FIB-SEM (correlative light and electron microscopy (focused ion-beam-scanning electron microscopy) of Rab7 marked endosomes. In one set of experiment, we discovered that these Rab7 and CPE co-labeled structures are multivesicular endosomes and migrate to the cleavage furrow during cytokinesis. Using CLEM/FIB-SEM we focused on those vesicles that are in close proximity to the ingressing membranes and discovered that the multivesicular endosomes are tethered to the ingressing membranes of the cytokinetic furrow. Further analysis using CLEM-transmission electron microscopy showed that the intraluminal vesicles within the MVBs were being deposited at the cleavage furrow in close proximity to the ingressing membranes, perhaps providing a source of membrane components for the newly formed and expanding plasma membrane of meiotically dividing cells.
2. As suggested by the reviewer, we evaluated meiosis in Rab7 mutants and dominant negative Rab11 mutants. We find between 15-25% of the spermatocytes fail to undergo meiosis in Rab7 mutant indicating a small but significant role for Rab7 in meiotic cytokinesis. Our observations show that CPE positive endosomes continue to form and cycle to the furrow in the Rab7 mutant indicating existence of redundant mechanisms

(Movie S13). Rab11 mutant are embryonic lethal and therefore we used conditional expression of dominant negative Rab11 mutants and discovered significant cytokinesis defect in them (Figure S7).

Another more technical concern is that the authors described cloning most of their transgenes into UAST vectors, which are well known for NOT being expressed in germ cells (Rorth, MoD, 1998 and Deluca and Spradling, Genetics, 2018). Either it is a multiple typo throughout the text or it is major technical concern, because it would be hard to interpret most of the results based on these transgenes expression.

We have addressed the concern of the reviewer experimentally. The first generation pUAST vectors were shown to be poorly expressed in female germline, as a result Pernille Roth developed second generation UAS vector named pUASP that was optimized for expression in female germline (Roth P 1998;Deluca and Spradling, Genetics, 2018). However, it was shown that pUAST- driven construct express robustly in male gonads of Drosophila (Demarco R.S et al 2014, Methods, https://doi.org/10.1016/j.ymeth.2014.04.020 ; Helen White-Cooper, 2012, Spermatogenesis, https://doi.org/10.4161/spmg.19088). In addition, we have addressed the concern of the reviewer experimentally in the context of our studies, we have done the following experiment. We expressed pUAST-GFP, pUAST-PLCδ-PH-EGFP and pUASP-alpha-tubulin-GFP using *nos*-Gal4, *bam*-Gal4 and *chif*-Gal4 and see comparable expression in the male germ cells and have provided that data as Fig. S4 (A-K). We are therefore confident of our studies and conclusion drawn therein.

Revised manuscript (lines 364-386)

To verify the expression pattern of these Gal4 drivers in our experimental settings we have crossed *nos*-Gal4 (BDSC#4937) with various pUASP/pUAST GFP tagged proteins including pUASP-*alpha-Tubulin*-GFP, pUAST-EGFP, and pUAST-PLCδ-PHEGFP. As expected, *nos*-Gal4 expression is restricted to early germ cells present at the tip of the testis including germline stem cells (GSCs) and early spermatogonial cells (Fig.S4A, E & I). On the contrary, *bam*-Gal4 expression is strong just below the testis tip where spermatogonia and early spermatocytes are expected to be present (Fig.S4B, F & J). However, depending on the stability of the expressed protein *bam*-Gal4 mediated expression is either restricted to spermatogonia and early spermatocytes (eg. PLCδ-PHEGFP, Fig.S4J) or persisted through later stages including early, later spermatocytes and even in spermatids (eg. *Alpha tubulin* and EGFP, Fig.S4B & F). Expression of *chif*-Gal4 expression was detectable in spermatocytes (Fig.S4C) but more strongly in elongated spermatids (Fig.S4G & K). Interestingly, As shown in Fig.3, we observed varying degree of rescue, depending on the germ cell differentiation stage at which UAS-CPES was expressed. The *nos*-Gal4 dependent expression of UAS-CPES partially rescued both meiotic cytokinesis (Fig.3C and N) and spermatid polarity (Fig.3G) however, individualizing spermatids lacked cystic bulges and waste bags indicating individualization was not fully rescued (Fig.3K). The *bam*-Gal4 dependent expression of UAS-CPES in transit-amplifying cells completely rescued meiotic cytokinesis (Fig.3D, and N) and spermatid polarity, and individualizing spermatids contained cystic bulges and waste bags (Fig.3H and L). In contrast, only partial rescue of meiotic cytokinesis was observed when UAS-CPES was expressed in spermatocytes using *chif*-Gal4 (Fig.3E & 3N). Although spermatid polarity was completely rescued, the individualizing spermatids largely lacked cystic bulges and waste bags indicating that individualization was defective (Fig.3I and M).

Along the same line, it is very surprising (but possible) to have a rescue with the bam-Gal4 driver and not with nanos-Gal4. bam-Gal4 is a weak driver with a narrow expression window, whereas nanos has wider and stronger expression in germ cells. However, there are nanos-Gal4 transgene insertions, which show a variegated (and very variable) expression in testis. I would advise the authors to test several nanos-Gal4 insertions to strengthen these surprising results and to use a UASp-GFP to show the expression pattern of their drivers.

Nanos expression is highest in the stem cell compartment. Whereas *Bam* is expressed is restricted to the transit amplifying compartment of the spermatogonial cells. *Nanos* is predominant in the earlier compartments (stem cells to spermatogonial cells) of the developing testis while *Bam* appears at later stage (spermatogonial to spermatocyte stages).

We have tested several *nanos*-Gal4 lines and found that the driver we used in our study faithfully recapitulates the expression described for nanos. We do agree with the reviewer about being cautious using *nanos*-Gal4 since some of them do show broad expression as shown below in the experiment we performed where pUAST-GFP is driven by three different nanos-Gal4 (Fig. S4 L-N). Two of them (including the one used extensively in this study) were specific to stem cell compartment while the other (that was not used in our studies) was rather widely expressed.

<u>Revised manuscript (lines 364-418)</u>

To verify the expression pattern of these Ga4 drivers in our experimental settings we have crossed *nos*-Gal4 (BDSC#4937) with various pUASP/pUAST GFP tagged proteins including pUASP-*alpha-Tubulin*-GFP, pUAST-EGFP, and pUAST-PLCδ-PHEGFP. As expected, *nos*-Gal4 expression is restricted to early germ cells present at the tip of the testis including germline stem cells (GSCs) and early spermatogonial cells (Fig.S4A, E & I). On the contrary, *bam*-Gal4

expression is strong just below the testis tip where spermatogonia and early spermatocytes are expected to be present (Fig.S4B, F & J). However, depending on the stability of the expressed protein *bam*-Gal4 mediated expression is either restricted to spermatogonia and early spermatocytes (eg. PLCδ-PH-EGFP, Fig.S4J) or persisted through later stages including early, later spermatocytes and even in spermatids (eg. *Alpha tubulin* and EGFP, Fig.S4B & F). Expression of *chif*-Gal4 expression was detectable in spermatocytes (Fig.S4C) but more strongly in elongated spermatids (Fig.S4G & K). Interestingly, As shown in Fig.3, we observed varying degree of rescue, depending on the germ cell differentiation stage at which UAS-CPES was expressed. The *nos*-Gal4 dependent expression of UAS-CPES partially rescued both meiotic cytokinesis (Fig.3C and N) and spermatid polarity (Fig.3G) however, individualizing spermatids lacked cystic bulges and waste bags indicating individualization was not fully rescued (Fig.3K). The *bam*-Gal4 dependent expression of UAS-CPES in transit-amplifying cells completely rescued meiotic cytokinesis (Fig.3D, and N) and spermatid polarity, and individualizing spermatids contained cystic bulges and waste bags (Fig.3H and L). In contrast, only partial rescue of meiotic cytokinesis was observed when UAS-CPES was expressed in spermatocytes using *chif*-Gal4 (Fig.3E & 3N). Although spermatid polarity was completely rescued, the individualizing spermatids largely lacked cystic bulges and waste bags indicating that individualization was defective (Fig.3I and M).

Fig.3. Genetic dissection of spatiotemporal role of CPES in spermatogenesis. *(A) Schematic illustration of early germ cell differentiation and stage specific Gal4 expression patterns. (B-E) Phase contrast microscopy of round spermatids in live testis squash preparations. (F-I) Early elongating spermatids immunostained for beta tubulin primary antibody (Red) and DAPI for DNA (blue) (J-M) Individualizing spermatids immunostained for cleaved caspase DCP-1 antibody*

(Red) and DAPI for DNA(Blue). (B, F and J) C587-Gal4 drives the expression of UAS-CPES in cyst cells of cpes mutants. (C, G and K) Nanos-Gal4 drives the expression of UAS-CPES in GSCs and spermatogonia. (D, H & L) bam-Gal4 drives the expression of UAS-CPES in 4-16 cell stages of spermatogonia and spermatocytes (E, I & M) chif-Gal4 drives the expression of UAS-CPES in early spermatocytes. (N) Measurement of cytokinetic defects in testis squash preparations in cyst and germ cell specific rescue samples. Round spermatid count from 4 independent experiments for C587-Gal4 >UAS-CPES (n=500), 6 independent experiments nos-Gal4 >UAS-CPES (n=2500), 5 independent experiments for bam-Gal4 >UAS-CPES (n=2500), and 6 independent experiments for chif-Gal4 >UAS-CPES (n=3000). (O) Male fertility test where individual male from each of the UAS-CPES rescue experiment was crossed with 3 wild type females. The vials which produced at least 5 pupae or progeny were counted as fertile and plotted as percent fertility compared to wild type males. (P-S) Whole mount immunostaining followed by confocal imaging of genomic CPES flies where CPES is tagged with V5 at the C-terminus (red). GM130 is a cis-Golgi marker (green) and DAPI (blue) stains the DNA.

Fig.S4. Cell type specific expression pattern of various UAS and Gal4 constructs in Drosophila testis. *(A-) Whole mount Drosophila testis immunostained GFP with anti-GFP antibody (Green), DAPI stains DNA (Blue). (A) nanos-Gal4 (nos) drives the expression of pUASP-αTubulin-GFP (B) bam-Gal4 drives the expression of pUASP-alphaTubulin-GFP (C) chiffon-Gal4 drives the expression of alpha-Tubulin-GFP (D) C587-Gal4 drives the expression of alpha-Tubulin-GFP (E) nanos-Gal4 (nos) drives the expression of pUAST-EGFP (F) bam-Gal4 drives the expression of pUAST-EGFP (G) chiffon-Gal4 drives the expression of pUAST-EGFP (H) C587-Gal4 drives the expression of alpha-Tubulin-GFP (I) nanos-Gal4 (nos) drives the expression of pUAST-PLCδ-PH-EGFP (J) bam-Gal4 drives the expression of pUAST-PLCδ-PH-EGFP (K) chiffon-Gal4 drives*

the expression of pUAST-PLCδ-PH-EGFP (L-N) Verification of various nanos-Gal4 drivers and their expression pattern. BDSC#4937 (used in this study) and BDSC#64227 specifically express transgene in GSC and early spermatogonia compared to BDSC#32563 which expresses more broadly.

Rev. 4:

This paper reports on the discovery that CPE, a fatty acid, is required for cytokinesis in male meiosis. The conclusions are supported by the analysis of CPE synthase mutant, which is male sterile. The authors show some spermatogenesis and fertility defects in this mutant (Figures 2 and 3) and the strongest part of the paper. They have also effectively used the UAS / Gal4 system to control expression of cpes transgenes. The paper does not provide evidence for understanding how this lipid species promotes cytokinesis. Thus, this paper is mostly descriptive and lacks mechanistic insights. I also have concerns with the significance of the data in Figures 4, 6 and 7, as described below.

1. The descriptions of Figures 2 and 3 in the text could be improved. Why was each antibody used, such as Caspase? Note that the rationale for each antibody is described nicely for Figure 4 (eg lines 397-402). Some Figures (2-5) need legends on the figures panels to show what each color is. In Figure 2 and 3 the red represents different antibodies and this should be clear. This is done nicely in Figures 6 and 7.

We apologize for the ambiguity. We have now provided a rationale for the use of individual antibodies for figures 2 and 3. We have expanded on the legends to clarify the details of antibodies. Accordingly, the following changes have been incorporated.

Revised manuscript:

Description for the Vasa antibody (lines 180-181)

Immunostaining of *cpes* mutant testis with antibody to Vasa protein, a germ cell specific conserved RNA helicase [16], showed accumulation of germ cells at the tip of the testis (Fig. 2B)

Description for the caspase antibody (lines 249-255)

Activated caspases have a non-apoptotic role in cystic bulge and the waste bag of individualizing spermatids and are essential for successful spermatid individualization [27]. Immunostaining of wild type testis with cleaved *Drosophila* Caspase 1 (DCP-1) antibody showed elongated spermatids undergoing individualization that contained cystic bulges and waste bags (Fig.2J). In contrast, *cpes* mutant testis showed defectively elongated spermatids and was devoid of cystic bulges and waste bags indicating failure in spermatid individualization (Fig.2K).

For Figures 2 and 3 we have now included a description for each of the antibodies in the legends.

Figure 2 (lines 207-232)

Fig.2. Cytological, immunofluorescence and sphingolipid analyses in cpes mutants.

(A-C) Whole mount of Drosophila testis immunostained for germ cell specific Vasa protein primary antibody and Alexa Fluor 488 secondary antibody (Green) and DNA with DAPI (Blue) to visualize various stages of germ cells including stem cells, spermatogonia and spermatocytes (A) w^{1118}*, (B) cpes mutant and (C) Tub-Gal4>UAS-CPES rescue. (D-F) Phase contrast microscopy of live testis squash preparations to visualize round/onion stage spermatids (D)* w^{1118}*, (E) cpes mutant and (F) Tub-Gal4>UAS-CPES rescue. (G-I) Testis squash preparation followed by fixation and immunostaining to visualize early elongating spermatids. Arrow indicates spermatid head stained with DAPI (Blue) for DNA. The beta tubulin primary antibody and Alexa Fluor 568*

*conjugated secondary antibody (Red) were used for immunostaining spermatid tails (axonemes), w^{1118} (G), cpes mutant (H) and Tub-Gal4>UAS-CPES rescue (I). (J-L) Whole mount Drosophila testis immunostained with cleaved caspase specific DCP-1 primary antibody and Alexa Fluor 568 secondary antibody (Red) to visualize elongated and individualizing spermatids. DAPI stains the DNA (Blue). Cystic bulges are shown with arrows and waste bags with arrow heads (J) w^{1118} (K) cpes mutant and (L) Tub-Gal4>UAS-CPES rescue. (M) Quantification of meiotic cytokinesis defects in cpes mutants. Round spermatid count from 3 independent experiments for W^{1118} (n=1600), cpes (n=300) and from 4 independent experiments for tubulin-Gal4 >UAS-CPES rescue (n=2000). (N-P) Sphingolipid analysis in lipid extracts of dissected testis, wild type testis (WTTS); cpes mutant testis (CPMT) and bam-Gal4>UAS-CPES Rescue testis (ReTS). (N) Comparison of CPE and its subspecies between w^{1118}, cpes mutant and ReTS. (O) Comparison of ceramide and its subspecies between w^{1118}, cpes mutant and ReTS. (P) Comparison of hexosylceramide subspecies between w^{1118}, cpes mutant and ReTS. Statistical significance was calculated using mean, standard deviation (SD) and N in Prism 8. The 2way ANOVA multiple comparison was used to calculate P values where ****$P \leq 0.0001$; ***$P \leq 0.001$; **$P \leq 0.01$; *$P \leq 0.05$ and ns $P > 0.05$. Three independent biological replicates were taken for each sample and 250 pairs of testes were used for lipid extraction from each biological replicate.*

Figure 3 (lines 387-405)

Fig.3. Genetic dissection of spatiotemporal role of CPES in spermatogenesis. *(A) Schematic illustration of early germ cell differentiation and stage specific Gal4 expression patterns. (B-E) Phase contrast microscopy of round spermatids in live testis squash preparations. (F-I) Early elongating spermatids immunostained for beta tubulin primary antibody (Red) and DAPI for DNA*

(blue) (J-M) Individualizing spermatids immunostained for cleaved caspase DCP-1 antibody (Red) and DAPI for DNA(Blue). (B, F and J) C587-Gal4 drives the expression of UAS-CPES in cyst cells of cpes mutants. (C, G and K) Nanos-Gal4 drives the expression of UAS-CPES in GSCs and spermatogonia. (D, H & L) bam-Gal4 drives the expression of UAS-CPES in 4-16 cell stages of spermatogonia and spermatocytes (E, I & M) chif-Gal4 drives the expression of UAS-CPES in early spermatocytes. (N) Measurement of cytokinetic defects in testis squash preparations in cyst and germ cell specific rescue samples. Round spermatid count from 4 independent experiments for C587-Gal4 >UAS-CPES (n=500), 6 independent experiments nos-Gal4 >UAS-CPES (n=2500), 5 independent experiments for bam-Gal4 >UAS-CPES (n=2500), and 6 independent experiments for chif-Gal4 >UAS-CPES (n=3000). (O) Male fertility test where individual male from each of the UAS-CPES rescue experiment was crossed with 3 wild type females. The vials which produced at least 5 pupae or progeny were counted as fertile and plotted as percent fertility compared to wild type males. (P-S) Whole mount immunostaining followed by confocal imaging of genomic CPES flies where CPES is tagged with V5 at the C-terminus (red). GM130 is a cis-Golgi marker (green) and DAPI (blue) stains the DNA.

We have now expanded on the text to describe the defects observed in Figures 2 and 3. In the figures we have highlighted the cystic bulges (CB) with arrows and noted the absence of deficiency of CB in the mutant testis. We have also expanded on the spermatid polarity defect observed in hSMS1 rescue in *cpes* mutants. The following changes have been made in the text. The following section "**CPE deficient males are sterile and show defects in male meiotic cytokinesis, spermatid polarity and individualization" (Pages 8-13)** has been rewritten to provide better clarity as advised by the reviewer.

Similarly, the section on "**Transcriptomic and genetic dissection of spatiotemporal role of CPES in spermatogenesis**" in pages (14-20) has been reorganized and rewritten based on comments of reviewer 1 and 4.

3. A little more description of the cpes mutant would help. Although published, it would help the reader to know if the homozygotes have a viability defect (Flybase lists the males as "escapers", which suggests most homozygotes die).

In the introduction we have now included summarizing the defects observed in the cpes mutants as described by us previously including the pupal and perinatal lethality (Kunduri et al., PNAS, 2018). The following changes have been incorporated in the text.

Revised manuscript: (lines 88-93)

CPE is synthesized in the luminal compartment of trans Golgi by ceramide phosphoethanolamine synthase (CPES) (Fig.1A). Previously, we have shown that *cpes* null mutants show significant late pupal lethality during development and only about 25% of mutants survive to adulthood. About 60% of *cpes* mutant adults have dorsal closure defects and all the mutant males are sterile. Aged *cpes* mutant adults display light inducible seizures and paralysis due to defective cortex glia [15].

4. Does the bam-Gal4 driven expression show complete rescue? From the description on pg 13 this is not clear.

>Yes. Bam-Gal4 driven CPES completely rescues all the phenotypes observed in the male reproductive system in flies. We have now clarified this in the text.

Revised manuscript: (lines 380-382)

The *bam*-Gal4 dependent expression of UAS-CPES in transit-amplifying cells completely rescued meiotic cytokinesis (Fig.3D, and N) and spermatid polarity, and individualizing spermatids contained cystic bulges and waste bags (Fig.3H and L).

5. What is the frequency of cytokinesis failure (Figures 2-4).

The frequency of cytokinesis failure was now quantitated and shown in Fig.2M, Fig.3N, and Fig.S5D.

6. The importance of the data in Figure 4 to the overall story is not clear. Negative results with these kind of experiments is not very informative. Even the rescue with closely related species does not add much to the paper. This is perhaps shown by the absence of a significant conclusion at the end of this section (line 380). This Figure could be deleted or a supplementary figure.

We agree and thank the reviewer for the suggestion. Since the tick CPES did not rescue and we have not investigated the gene product in depth we have removed it from the revised manuscript. Also, we have now moved the data from rescue experiments using mosquito, silkworm CPES and human SMS1 to supplemental section (Fig. S5).

7. Line 326: how is it known that tub-Gal4 overexpresses the transgenes. This terminology is also a problem given the expressed gene is from another species.

We thank the reviewer for the suggestion, and we have now removed the word "overexpress. Instead, we have written it as "ubiquitously expressed" ……….

The flybase description of the expression pattern lists this Gal4 driver to be ubiquitously expressed from embryonic to adult stages (https://flybase.org/reports/FBtp0002651)

Revised manuscript (lines 444-455)

To this end, UAS-hSMS1 was ubiquitously expressed using tubulin-Gal4 in *cpes*, the adult male testes were dissected, and live testis squash preparations were observed using phase contrast microscopy. However, as shown in Fig.S5A, hSMS1 overexpression did not rescue male meiotic cytokinesis. Further, immunostaining analysis showed that spermatid polarity was also not rescued (Fig.S5E) suggesting an important role for the CPE head group in spermatogenesis. We next investigated whether expression of other insect derived CPES could rescue *cpes* mutant phenotypes. To test this, we ubiquitously expressed *Aedes aegypti* (Yellow fever mosquito, amino acid sequence shows 60% identity and 77% similarity), and *Bombyx mori* (domestic silk moth, amino acid sequence shows 43% identity and 58% similarity) derived UAS-CPES homologs in *cpes* mutant background using tubulin-Gal4. As shown in Fig.S5 mosquito and silk moth CPES were able to completely rescue defects in meiotic cytokinesis (Fig.S5B-D) and spermatid polarity (Fig.S5F and G respectively).

8. Figure 5 shows that there is a failure to complete furrow ingression, and there are some defects in the organization of the spindle midzone. The midzone defects are mild and this section (lines 410-413) could use some quantification, such as how often this phenotype is observed or is there any variation.

We thank the reviewer for the suggestion and have now quantified the data in the original Figure 5 (New figure 4B)

Revised manuscript (line 551-556)

Quantification of this behavior suggested that more than 80% of the spermatocytes showed detachment of the contractile ring and central spindle (Fig.4B). Other minor variations included the contractile ring detaching from the plasma membrane, contractile ring sliding and dumping to one end and the contractile ring and central spindle being destabilized simultaneously at one end (Fig.4B). Expression of UAS CPE in germ cells was sufficient to rescue this phenotype (Movie S4, Fig.4A bottom panel).

9. The result in Figure 6A-D is significant showing that PlyA2 localization is affected by the cpes mutant. The rest of the experiments in Figures 6 and 7, however, are descriptive of endosomes behavior and are do not directly test their function in cytokinesis. It is interesting that proteins like Rab7 and Rab11 localize to the midzone. From these results, however, it is not clear this is important for cytokinesis or if this localization depends on cpes. This lessens the significance of these results.

Reviewers 1, 3 and 4 have suggestions and concerns regarding the delivery of CPE via the endosomes. In response to the comments and suggestions of the reviewers, we have performed experiments that have provided more insight into the role of endosomes in delivery of CPE to the ingressing furrow. Briefly,

1. We have performed CLEM/ FIB-SEM (correlative light and electron microscopy (focused ion-beam-scanning electron microscopy) of Rab7 marked endosomes. In one set of experiment, we discovered that these Rab7 and CPE co-labeled structures are multivesicular endosomes and migrate to the cleavage furrow during cytokinesis. Using CLEM/FIB-SEM we focused on those vesicles that are in close proximity to the

ingressing membranes and discovered that the multivesicular endosomes are tethered to the ingressing membranes of the cytokinetic furrow. Further analysis using CLEM-transmission electron microscopy showed that the intraluminal vesicles within the MVBs were being deposited at the cleavage furrow in close proximity to the ingressing membranes, perhaps providing a source of membrane components for the newly formed and expanding plasma membrane of meiotically dividing cells.

2. As suggested by the reviewer, we evaluated meiosis in Rab7 mutants and dominant negative Rab11 mutants. We find between 15-25% of the spermatocytes fail to undergo meiosis in Rab7 mutant indicating a small but significant role for Rab7 in meiotic cytokinesis. Our observations show that CPE positive endosomes continue to form and cycle to the furrow in the mutant indicating existence of redundant mechanisms (Movie S13). Rab11 mutant are embryonic lethal and therefore we used conditional expression of dominant negative Rab11 mutants and discovered significant cytokinesis defect in them (Figure S7).

In compliance with data protection regulations, you may request that we remove your personal registration details at any time. (Use the following

URL: https://www.editorialmanager.com/pbiology/login.asp?a=r). Please contact the publication office if you have any questions.
