# Supplementary material for: Delivery of ceramide phosphoethanolamine lipids to the cleavage furrow through the endocytic pathway is essential for male meiotic cytokinesis
Source: PLoS Biol. 2022 Sep 28;20(9):e3001599. doi: 10.1371/journal.pbio.3001599 (PMC9550178; doi:10.1371/journal.pbio.3001599)
Supplement: Attachment — Submitted filename: Response to reviewer.docx [file pbio.3001599.s040.docx]

**Response to Reviewers**

**Response to Reviewers**

We thank the reviewer for comments and suggestions that have improved the quality of the manuscript considerably. We hope the changes we have incorporated following their suggestions and the clarifications we have provided in our response to the reviewer would provide sufficient clarity and elevate the quality of the work for publication in PLOS Biology.

In this revised submission, we have incorporated the following changes to the manuscript in response to the reviewer’s comments and suggestions.

1. Figure 4 has been expanded. We have performed quantification of the rate and magnitude of cytokinetic furrow ingression in the control, mutant and rescue dividing spermatocytes (Fig. 4C and D). We have replaced PLCdelta PH-EGFP images for cpes with more representative examples in Fig.4E as suggested by the reviewer. Statistical analyses of fluorescence intensity of reporters for PI(4,5)P_2_ and PI(3,4,5)P3 (Fig. 4F and Fig.4H).
2. Figure 5, panels N, O and P were replaced with a more representative example.
3. Figure 6G: Statistical analyses of PlyA2, Rab7 and PlyA2-Rab7 endosomal localization at the furrow. Fig. 6Q: Statistical analyses of PlyA2, Rab11 and PlyA2-Rab11 endosomal localization at the furrow.
4. Figure 8: The model has been redrawn upon the advice of the reviewer.
5. Figure S7: In response to reviewer’s suggestions, we have included a new Figure S7 that provides representative figures for microtubule arrangement and chromosomal alignments during metaphase-telophase stages of meiosis I in dividing spermatocytes. Accordingly other supplemental figures have been relabeled.
6. Figure S8: The original Fig.S7 is now Fig.S8 and has been reorganized. The new figure includes analyses of cytokinetic defects in Rab35 mutants (Panels C and F). The remainder of the panels have been accordingly relabeled.
7. Figure S9: This is a new figure. This figure examines the fraction of PlyA2-mCherry positive endosomes at the furrow in control, Rab7, Rab11 DN and Rab35 mutants. These experiments were done in response to reviewer’s suggestions.
8. Figure S10: This is a new figure. This figure examines spermatogenesis in Rab35 mutants and Rab35 localization in spermatocytes, early spermatids, and cyst cells.
9. Movie S2: We have expanded the timeline of the same old Movie S2 to show Sqh GFP in controls where it displays linear structures like those observed in *cpes* mutants.
10. Movie S13: New Movie – Live imaging of control spermatocytes treated with PlyA2 mCherry.
11. Movie S14: New Movie - Live imaging of Rab11 DN spermatocytes treated with PlyA2 mCherry.
12. Movie S15: New Movie - Live imaging of Rab35 mutant spermatocytes treated with PlyA2 mCherry. Others have been accordingly relabeled.
13. We have incorporated all other editorial and other corrections suggested by the reviewer including updated bibliography, corrections to bibliography and edits suggested to the text.

**Response to Reviewer Comments:**

1- A more detailed description of the cytokinetic defects is needed. Based on the fluorescent Feo + sqh movies, the authors should provide the rate and magnitude of furrow ingression for individual cells in control vs mutant situations. From movie S3, it seems that the actin ring detaches from the plasma membrane in most instances, which is not well captured in fixed samples (Fig. 4B).

We thank the reviewer for the suggestions and apologies for not being clear on Fig.4B quantification, whether it is fixed or live sample. Fig.4B is a quantitative representation of live spermatocytes undergoing cytokinesis. Yes, it is also our observation that the actomyosin ring detaches from the plasma membrane in most instances in representative Movies S3. However, we believe it is a secondary effect since we see disengagement of central spindle and contractile ring first and in the end the contractile ring detaches from plasma membrane. Fig.4B represents first observable phenotype in *cpes* mutant spermatocytes undergoing division. Further, under similar experimental settings with intact cysts, cytokinetic defects were negligible in control cells and therefore we did not include quantification for the same. We have now not only explicitly mentioned this defect in the text but also in Fig. 4B legends. Following the advice of the reviewer, we have now measured the rate and magnitude of furrow ingression and compared the control and the mutant dynamics (Fig. 4C) and graphed a representative comparison (at 40 min) of the furrow diameters in Fig. 4D. Accordingly, the text is now edited to include these observations and findings.

These changes are incorporated into the text as described below.

**Revised Submission (lines 476-489)**

Interestingly, in *cpes* mutants, the initial assembly of central spindle and actomyosin ring occurred normally (Movie S3) however, central spindle and actomyosin ring constriction became uneven since there was barely any furrow ingression and led to substantial disengagement between the contractile ring and the central spindle during meiosis (Fig.4A, C & D). As a result, in the mutants the actomyosin ring either detached from the plasma membrane and/or the central spindle and actomyosin ring disassembled prematurely (Movie S3). Quantification of this behavior in live *cpes* mutant spermatocytes suggested that more than 80% of the spermatocytes showed disengagement of the contractile ring and central spindle prior to contractile ring detachment from the plasma membrane (Fig.4B, Movie S3). Other minor variations included contractile ring detachment from the plasma membrane prior to central spindle and contractile ring disengagement, contractile ring sliding and dumping to one end and the contractile ring and central spindle being destabilized simultaneously at one end (Fig.4B). Under similar experimental settings in intact cysts cytokinetic defects are negligible. Expression of UAS-CPES in germ cells was sufficient to rescue this phenotype (Movie S4, Fig.4A, C & D).

**Accordingly, Fig.4 legends has also been edited as follows: (lines 490-517)**

***Fig.4. Aberrant central spindle behavior but not localization of PIPs at the plasma membrane is responsible for meiotic cytokinesis defect in cpes mutants.*** *(A, C & D) Snapshots of live spermatocytes undergoing meiosis I cytokinesis. In horizontal view (left), one slice of a z-stack is shown. In cross-sectional view across cleavage furrow, maximum intensity projection image is shown (right). Control spermatocytes (top row), cpes mutant spermatocytes (middle row) and tubulin-Gal4>UAS-CPES rescue (bottom row). (A) Spermatocytes expressing myosin II regulatory light chain tagged to GFP under its own promoter (sqh) that localizes to contractile ring and mCherry tagged fascetto (feo) under the control of Ubi-p63E promoter that binds to central spindle microtubules. (B) Quantification of spindle organization defects in live cpes mutant spermatocytes. First observable phenotype was measured and plotted. Central spindle (CS) detachment from contractile ring (CR), detachment of CR from plasma membrane (PM), CR slides on PM and CS and CR destabilizing from end. (C) Quantification of rate and magnitude of cytokinetic furrow ingression using ImageJ and Prism9. Cytokinetic furrow diameter was measured as a function of time in dividing spermatocytes from control (n=12), mutant (n=10) and rescue sample (n=9). Statistics were performed using 2way ANOVA multiple comparisons in Prism 9. (D) A representative bar diagram for cytokinetic furrow diameter at 40 min post furrow ingression in C. (E) Spermatocytes expressing GFP tagged pleckstrin homology (PH) domain of PLCD (UAS-PLCδ-PH-EGFP) under the control of Tub-Gal4 that localizes to plasma membrane in the presence of phosphatidylinositol-4,5-bisphosphate (PI(4,5)P2) and Ubi-p63E-feo-mCherry. (F) Fluorescence intensity measurements for live spermatocyte cysts expressing Tub-Gal4> UAS-PLCδ-PH-EGFP, control (n=15 cysts), cpes mutants (n=18 cysts) and rescue (n=20 cysts). Statistics were performed using ordinary one-way ANOVA multiple comparisons in Prism 9. (G) Spermatocytes expressing GFP tagged PH domain of steppke/Grp1 under the control of alpha Tub84B promoter (tGPH) that localizes to plasma membrane in the presence of phosphatidylinositol-3,4,5-triphosphate (PI(3,4,5)P3) and Ubi-p63E-Feo-mCherry. (H) Fluorescence intensity measurements for live spermatocyte cysts expressing tGPH. control (n=8 cysts), cpes mutants (n=12 cysts) and rescue (n=12 cysts). Statistics were performed using ordinary one-way ANOVA multiple comparisons in Prism 9.*

In addition, Feo is present on linear structures in early meiosis in the cpes mutant (movie S3) but not in controls (movie S4): how are the spindle and the microtubules in the cpes mutant?

A basic description of the chromosomes is also needed: do chromosomes struggle to align properly (I noticed BUBR1 in Fig. S6E)?

We thank the reviewer for the suggestion, and if we understood correctly, reviewer was asking about the green linear structures corresponding to Sqh-GFP present in cpes mutants but not in control. If so, we see these linear structures even in control spermatocytes in earlier time points of meiosis. We have now included the same movie S2 starting with few time points earlier. Further, we carefully observed, documented, and compared the state of microtubules and the chromosomes during meiosis in the control and the mutants. We do not see significant differences in the dynamics of microtubules and chromosomes during metaphase and anaphase spermatocytes between control and *cpes* mutants. However, we do see microtubule density and organization related defects in telophase spermatocytes. This data is now included as a separate new supplemental figure S7.

We have made edits to the text to include information gleaned from the experiments described in Figure S7.

**Revised Submission (lines 455-460)**

the accurate prediction of altered pathways in *cpes* mutants. Notably, immunostaining analysis of testis squash preparations with alpha tubulin antibody did not show consistent differences in astral spindle organization and chromosomal alignment in metaphase and anaphase spermatocytes (Fig. S7A-C and G-H). However, spindle microtubules and central spindle (marked by feo-Cherry) were less dense and disorganized in early to late telophase spermatocytes indicating spindle behavior is affected in *cpes* mutants during cytokinesis (Fig. S7D-F and J-L).

**and the corresponding legend (lines 1623-1627)**

*Fig.S7. Analysis of spindle microtubules and chromosomal DNA in testis squash preparations.*

*Microtubules are immunostained with apha tubulin antibody and chromosomal DNA with DAPI. Confocal images of wild type spermatocytes in metaphase (A-C), anaphase (D), early telophase (E) and late telophase (F). Confocal images of cpes mutant spermatocytes in metaphase (G-I), anaphase (J), early telophase (K) and late telophase (L).*

2- The impact of the paper would be further increased if the authors could demonstrate that i) CPE is delivered by endosomes to the furrow membrane and ii) this is important for cytokinesis. In the present manuscript, there are only interesting correlative evidence. In this respect, the conclusion (line 639) is an overstatement.

First, is CPE locally enriched at the furrow MEMBRANE when Rab7 endosomes are in its vicinity in control cells? If Rab7 endosomes deliver CPE to the furrow for proper ingression, this would be expected.

We thank the reviewer for the suggestion. However, we have been unable to label the furrow membranes and observe it in the context of CPE delivery for couple of reasons. First addition of exogenously labeled PlyA2 strongly labels all the plasma membrane including cleavage furrow uniformly and hence specific spike in CPE at the furrow is challenging to detect with this tool. Secondly, we expressed PlyA2 cytosolically to see if newly added CPE is detectable on the furrow membranes, although this probe did detect those MVBs with significant loss of outer membrane where CPE is exposed to cytosol (as described in Fig.8B & C), but it did not detect furrow membranes specifically (our unpublished ongoing studies). We have even tried double labeling i.e., adding PlyA2 exogenously (PlyA-mCherry) to spermatocytes expressing PlyA2-EGFP cytosolically. Again, we see colocalized endosomes at the furrow but not labeling of the furrow membranes (unpublished ongoing studies). Thus, we were limited to utilizing labeled actomyosin ring as a proxy for following the furrow and its neighborhood during meiosis and then performed FIB-SEM and FIB-SEM-TEM on specific spatio-temporally captured samples with the hope of obtaining highly correlated images of vesicle release, membrane ingression and any additional features that we could observe. Our endeavors provided us some of the most insightful events occurring at the ingressing furrow. Unfortunately, this method still does not allow us to observe in real time the delivery of CPE to an easily identifiable ingressing membrane at the furrow. Hopefully, future endeavors might allow us to devise methods that would make such observations possible.

We have modified the concluding sentence to indicate that Rab7 and Rab11 function in delivery of CPE to the furrow. Accordingly, the sentence now reads as follow

**Revised Submission (lines 587-589)**

Overall, the endocytosis of CPE and targeting to the ingressing cleavage furrow *via* Rab7 and Rab11 marked endosomes indicates their importance in delivery of lipids to the ingressing membranes of the furrow.

Second, the authors should try to find a mutant situation where the CPE endosomes are not properly localized to the furrow. Although Rab7 mutants failed to show this, what does happen in the Rab11 DN situation (this is only touched in the discussion)?

Rab11 DN and spermatocytes and PlyA2-mCherry experiment

Based on movies, how does the CPE probe (especially at the PM of the furrow) exactly behave during cytokinesis in the Rab7 mutant or in the Rab11 DN mutant (beside single movies, quantifications are needed)?

We thank the reviewer for the suggestion and as suggested by the reviewer, we have now performed live imaging experiments using Rab7 mutants, Rab11 DN situation with PlyA2-mCherry labeling. We have observed and quantitated the PlyA2-mCherry behavior in the control, Rab7 mutant and in the Rab11 DN mutant background. In short, we find that there is a significant reduction in the number of PlyA2 positive endosomes at the cleavage furrow in the Rab11 DN negative expressing spermatocyte indicating a need for Rab11 in localization of PLyA2 endosomes at the furrow. Also, consistent with our other findings we do find that in Rab7 mutants, PlyA2-mCherry continued to be endocytosed and targeted to the cleavage furrow. The new experiments are now documented as follows:

We have now included a new figure S9 that records the PlyA2-mCherry positive endosomes in dividing spermatocytes, recording of total PlyA2-mCherry positive endosomes, furrow specific PlyA2-mCherry positive endosomes and quantitation of the endosomes in the furrow from several dividing spermatocytes in the control, *rab7*, Rab11 DN and *rab35* mutant (in response to reviewer suggestion addressed in the paragraph to follow) backgrounds. Accordingly, the following changes have now been incorporated into the revised manuscript.

**Revised submission (lines 602-614) - for Rab11 DN experiments cytokinetic defects.**

Consistent with previously published results [22, 24], we found significant increase in cytokinetic defects in Rab11DN expressing gonads (Fig.S8B & F), confirming important roles for Rab11 mediated membrane trafficking during cytokinesis. To further investigate CPE positive endosome behavior during cytokinesis, we treated Rab11 DN expressing spermatocytes with PlyA2-mCherry and performed live cell imaging. Interestingly, compared to control spermatocytes (Fig.S9A-C & M), Movie S13), Rab11 DN expressing spermatocytes showed significant reduction in CPE positive endosome localization to cleavage furrow (Fig.S9D-F & M) and Movie S14). Further, cleavage furrow regressed following ingression in Rab11 DN expressing spermatocytes, Movie S14. These results suggest that normal Rab11 function is required for furrow specific localization of CPE containing endosomes. Cleavage furrow regression after complete ingression in Rab11 DN expressing spermatocytes indicates endosome mediated CPE addition may play an important role in late steps of cytokinesis perhaps by providing stability to the ingressed membranes.

**Revised submission (lines 647-653) - includes the data from PlyA2-mcherry experiments in Rab7 mutants.**

Consistent with this possibility, we observed that PlyA2-mCherry continued to be endocytosed and targeted to cleavage furrow in Rab7 mutants (Fig.S9J-L & M, Movie S16). However, presence of significant cytokinetic defects in Rab7 mutants indicate that normal Rab7 function is required for proper dynamics of CPE positive endosomes via as yet unknown mechanisms during cytokinesis. Taken together these genetic analyses indicate that Rab7, Rab11 and Rab35 functions are required for normal male meiosis cytokinesis, however, Rab7 and Rab11 functions are more directly linked to CPE positive endosome behavior during cytokinesis.

I also suggest that the authors should have a look to Rab35 mutants, to uncouple CPE endosomes from the furrow (YFP-Rab35 WT and DN flies are available). Indeed, i) Rab35 is a recycling endosome marker and is required for cytokinesis (many papers since 2006), ii) Rab35 controls MVB fusion to the PM (PMID 20404108), iii) Rab35 is a negative regulator of mTOR during myelin growth (PMID 32503983). This might be an interesting connection since TOR is the first hit in Fig. S6C and CPE mimics GalCer, which is a major sphingolipid in myelin sheath (as discussed, line 814).

We thank the reviewer for the suggestion and have undertaken studies to evaluate CPE endosomes in this mutant background.

Our experiments confirm that Rab35 mutants are semilethal and mutant male escapers were observed. Rab35 mutants display significant cytokinetic, spermatid polarity and individualization defects. Surprisingly, our live imaging experiments did not show strong plasma membrane and endosomal localization of Rab35 in dividing spermatocytes. However, it was enriched in the endosomes of the encompassing somatic cyst cells. We also did not find significant changes in the localization of the PlyA2-mCherry positive endosomes to the ingressing furrow indicating that Rab35 might have minimal role in localization of CPE to the furrow. We have provided a detailed description of our findings and possible implications in a new paragraph appended to the revised manuscript and provided below for the reviewer’s perusal.

**Revised manuscript (lines 615-634)**

Rab35 is another recycling endosomal marker that was shown to be essential for cytokinesis in mitotic cells [70]. A recent study showed that Rab35 mutant flies are semilethal and mutant male escapers were observed [71]. We performed cytological and immunostaining analysis on Rab35 mutant testes and found significant cytokinetic defects, spermatid polarity and individualization defects (Fig.S8C & Fig.S10A-D). Earlier studies have shown that Rab35 localizes to the plasma membrane and endosomal compartments in mitotic cells [70]. However, EYFP-Rab35 did not show clear plasma membrane or endosomal localization in immunostained spermatocytes, instead it was predominantly localized to cytosol (Fig.S10E). However, we did see its enrichment on endosomal compartment in cyst cells (Fig.S10E &F). Interestingly, in the live spermatocytes EYFP-Rab35 shows mitochondria like localization pattern in spermatocytes undergoing cytokinesis and nebenkern in round spermatids (Fig.S10G & H respectively). Nevertheless, we have investigated CPE positive endosome behavior in Rab35 mutant spermatocytes and found no significant difference in endocytosis and localization of CPE containing endosomes to the cleavage furrow (Fig.S9G-I & M, Movie S15). These results suggest that Rab35 may not be involved in cleavage furrow specific localization of CPE positive endosomes. However, we cannot rule out the possibility that Rab35 could act after CPE positive endosome translocation to the cleavage furrow, i.e., lipid recycling at the furrow. Another possibility could be that Rab35 is required for delivery of other lipids and or protein cargo such as Phosphatidylinositol 5 phosphatase (OCRL), oxidoreductase (MICAL1) etc., [23], independent of CPE, to facilitate late steps of cytokinesis in spermatocytes.

3- The absence of defects in PI(4,5)P2 distribution in cpes mutants should be quantified and analyzed in more detail.
First, it seems that PI(4,5)P2 distribution is not uniform in controls (less present at the furrow, compared to the pole, Fig. 4C) whereas PI(4,5)P2 is evenly distributed along the PM in cpes mutant.

Second, there is a striking increase of PI(4,5)P2 amounts (based on the PH-GFP probe) at the PM in mutants (Fig. 4C) with more membrane ruffles (movie S6). Is this true? In this case, would reducing PI(4,5)P2 (e.g. by reducing PI4P-5-kinase activity in an heterozygous Skittle mutant) rescue the cpes mutant phenotype?

We have now replaced the control PI(4,5)P2 image to represent uniform distribution along the PM. The apparent increase in PI(4,5)P2 levels at the poles or other parts of the images are due to cyst cell and/or neighboring spermatocyte PM integrations.
The increase of PI(4,5)P2 at the PM in mutants was more an exception of one dividing spermatocyte rather than a general occurrence. We apologize for overlooking this fact when we chose the sample. We have changed this panel with an image of a more representative spermatocyte. Heeding the reviewer’s suggestion, we have quantified both PI(4,5)P_2_ and PI(3,4,5)P_3_ distribution from several control, mutant and rescue spermatocytes. We do not observe a significant difference in the quantification of either of these two reporters in the plasma membrane between control and the mutants. While PI(4,5)P_2_ was decreased and PI(3,4,5)P_3_ was moderately increased in the rescue compared to the control or mutant, it did not have any impact on division or cytokinesis in the rescued spermatocytes. Similarly, membrane ruffles in Movie S6 are originating from the cyst membranes. We have seen such ruffles in some of the rescue membranes also, again they do not correlate with cytokinetic defects. These new findings have been appropriately incorporated in the manuscript and the following edits are notable.

**Revised submission (lines 490-556)**

***Fig.4. Aberrant central spindle behavior but not localization of PIPs at the plasma membrane is responsible for meiotic cytokinesis defect in cpes mutants.*** *(A, C & D) Snapshots of live spermatocytes undergoing meiosis I cytokinesis. In horizontal view (left), one slice of a z-stack is shown. In cross-sectional view across cleavage furrow, maximum intensity projection image is shown (right). Control spermatocytes (top row), cpes mutant spermatocytes (middle row) and tubulin-Gal4>UAS-CPES rescue (bottom row). (A) Spermatocytes expressing myosin II regulatory light chain tagged to GFP under its own promoter (sqh) that localizes to contractile ring and mCherry tagged fascetto (feo) under the control of Ubi-p63E promoter that binds to central spindle microtubules. (B) Quantification of spindle organization defects in live cpes mutant spermatocytes. First observable phenotype was measured and plotted. Central spindle (CS) detachment from contractile ring (CR), detachment of CR from plasma membrane (PM), CR slides on PM and CS and CR destabilizing from end. (C) Quantification of rate and magnitude of cytokinetic furrow ingression using ImageJ and Prism9. Cytokinetic furrow diameter was measured as a function of time in dividing spermatocytes from control (n=12), mutant (n=10) and rescue sample (n=9). Statistics were performed using 2way ANOVA multiple comparisons in Prism 9. (D) A representative bar diagram for cytokinetic furrow diameter at 40 min post furrow ingression in C. (E) Spermatocytes expressing GFP tagged pleckstrin homology (PH) domain of PLCD (UAS-PLCδ-PH-EGFP) under the control of Tub-Gal4 that localizes to plasma membrane in the presence of phosphatidylinositol-4,5-bisphosphate (PI(4,5)P2) and Ubi-p63E-feo-mCherry. (F) Fluorescence intensity measurements for live spermatocyte cysts expressing Tub-Gal4> UAS-PLCδ-PH-EGFP, control (n=15 cysts), cpes mutants (n=18 cysts) and rescue (n=20 cysts). Statistics were performed using ordinary one-way ANOVA multiple comparisons in Prism 9. (G) Spermatocytes expressing GFP tagged PH domain of steppke/Grp1 under the control of alpha Tub84B promoter (tGPH) that localizes to plasma membrane in the presence of phosphatidylinositol-3,4,5-triphosphate (PI(3,4,5)P3) and Ubi-p63E-Feo-mCherry. (H) Fluorescence intensity measurements for live spermatocyte cysts expressing tGPH. control (n=8 cysts), cpes mutants (n=12 cysts) and rescue (n=12 cysts). Statistics were performed using ordinary one-way ANOVA multiple comparisons in Prism 9.*

Previous studies have shown that phosphatidylinositols (PIP) play critical roles in somatic and meiotic cytokinesis [57]. Further, in mammalian (HeLa) cells undergoing mitosis, sphingomyelin-rich lipid domains in the outer leaflet of the cleavage furrow were required for accumulation of PI(4,5)P_2_ to the cleavage furrow which in turn was required for proper translocation of RhoA and progression of cytokinesis [10]. We wondered whether the distribution of PIP at the plasma membrane or at the cleavage furrow is altered in *cpes* mutants during meiosis. To investigate PIP distribution in *cpes* mutant membranes we used PI(4,5)P_2_ reporter UAS-PLCδ-PH-EGFP (UAS regulatory sequence drives expression of EGFP fused to the PH domain of human PLCdelta 1) and PI(3,4,5)P_3_ reporter tGPH (an alphaTub84B promoter drives the expression of EGFP fused to the PH domain of Steppke) [58-60]. The PI(4,5)P_2_ reporter PLCδ-PH-EGFP was shown to uniformly localize to the entire membrane including cleavage furrow in *Drosophila* spermatocytes [61]. Indeed, we have seen similar uniform distribution in both control and *cpes* mutant spermatocyte plasma membranes indicating normal distribution of PI(4,5)P_2_ (Movie S5 and S6, Fig.4E). Live imaging analysis on *cpes* mutant spermatocytes expressing PLCδ-PH-EGFP revealed normal cleavage furrow initiation, but progression of furrow failed due to the disconnect between central spindle and the furrow (Movie S6, Fig.4E). Expression of wild type CPES rescued this phenotype (Movie S7, Fig.4E). Of note, PLCδ-PH-EGFP localization to spermatocyte plasma membrane was significantly reduced upon loss of cyst cells although this acute reduction did not prevent spermatocytes from completing cytokinesis during meiosis 1. Fluorescence intensity measurements for PLCδ-PH-EGFP localization showed no significant difference between control, and *cpes* mutant cysts suggesting PI(4,5)P_2_ localization was not affected in *cpes* mutants (Fig.4F). Although, rescue cysts showed reduction in PI(4,5)P_2_ levels it did not correlate with the rescue phenotype. We next investigated the PI(3,4,5)P_3_ distribution in control meiotic spermatocytes using tGPH. Interestingly, we found that tGPH localized to plasma membranes and became significantly enriched at cleavage furrows (Movie S8, Fig.4G). Of note, even here, we have observed that plasma membrane localization and cleavage furrow enrichment of tGPH strictly depended on the intact cyst. While it is currently unknown if PI(3,4,5)P_3_ localization to the spermatocyte cleavage furrows has any impact on cytokinesis, a recent study has shown that cytohesin Steppke reduces tissue tension by inhibiting the actomyosin activity at adherens junctions and actomyosin network assembly is necessary and sufficient for local Steppke accumulation in *Drosophila* embryos [62]. Next, we wondered if cleavage furrow enrichment of PI(3,4,5)P_3_ is altered in *cpes* mutants. However, as shown in Movie S9, Fig.4G, PI(3,4,5)P_3_ localization to the cleavage furrow was not altered but again there was a disconnect between cleavage furrow and central spindle during cleavage furrow ingression. Expression of wild type CPES rescued this phenotype (Movie S10; Fig.4G). Further, fluorescence intensity measurements showed no significant difference between control and *cpes* mutant cysts suggesting PI(3,4,5)P_3_ distribution is not compromised in *cpes* mutants (Fig.4H). Overall, these observations suggest that aberrant spindle behavior but not the localization of PIPs to the plasma membrane or cleavage furrow, is responsible for meiotic cytokinesis defect in *cpes* mutants.

**Minor points**

1- Fig 4B: please provide quantifications in controls.

We thank the reviewer for suggestion, however, due to absence of significant cytokinetic defects in intact cysts of controls in our experiments we did not include this data. We have now included a sentence to note this observation in the text.

2- The picture in Fig. 5P does not reflect the quantification for Rab11/CPE colocalization in Fig 5Q. Either the picture is not representative, or the quantification is not accurate.

We apologize for the error. The picture was not representative. We have replaced the panel (5N, 5O and 5P) with a more representative one.

3- Fig. 6: please provide movies and quantification of PlyA2 in cells that do no express Rab proteins (see major point #2).

We have now provided movies and quantification of PlyA2 in cells that do not express Rab11 (Fig.S9A-C & M), Movie S13), Rab7 (Fig.S9D-F &M, Movie S16) and Rab35 mutants (Fig.S9J-L & M, Movie S15) mutants. As the reviewer has indicated these experiments and new data were performed and incorporated in response to reviewer’s suggestions in major point #2.

4- Fig. 8E: the fusion of a small endosome to an MVB is misleading. Endosomes first fuse together to form large early endosomes that eventually mature into MVBs with ILVs.

We have now redrawn the model to include large early endosomes that mature into MVBs (Fig. 8E)

5- References should be improved. Some cited reviews are good but outdated, original papers are not always cited or previous published results are not associated with references. For instance, a paper that seems important to cite and discuss in the light of the present study is PMID 18804373 (line 84).

We have revised the manuscript to include newer reviews, included several original references and correctly incorporated a few references that were not correctly associated with the text.

6- Line 310: to validate the conclusion, it would be important to provide a quantification of the ceramide levels (as in Fig. 2O) upon ceramidase expression.

This is a valid point and we put substantial effort to estimate sphingolipids in this genetic background. We obtain very few flies that express ceramidase (using the Vasa-Gal4 line) in the *cpes* mutant background. We need between 800-1200 testes to estimate the sphingolipids in these samples. In the three attempts we made, we obtained fewer than 200 ceramidase expressing *cpes* mutant flies and were thus not able to estimate sphingolipids in these samples. However, in the past we and others (to whom we have sent UAS-ceramidase flies) have expressed the ceramidase gene using other Gal4 lines and successfully estimated the sphingolipid content in those conditions. In all these conditions expression of ceramidase was always associated with decreased ceramide levels. Hence, we only mention it without alluding to the actual sphingolipid content in the manuscript.

7- Change PIP2 and PIP3 to PI(4,5)P2 and PI(3,4,5)P3, respectively, for clarity.

We thank the reviewer and have changed PIP2 to PI(4,5)P_2_ and PIP3 to PI(3,4,5)P_3_ throughout the manuscript.

8- Line 642: missing BDSC#

We have now included the. Missing BDSC#23261 (line 592)
